# MLBRSA: Multi-Learning-Based Reptile Search Algorithm for Global Optimization and Software Requirement Prioritization Problems

**DOI:** 10.3390/biomimetics8080615

**Published:** 2023-12-15

**Authors:** Jeyaganesh Kumar Kailasam, Rajkumar Nalliah, Saravanakumar Nallagoundanpalayam Muthusamy, Premkumar Manoharan

**Affiliations:** 1 Department of Artificial Intelligence and Data Science, M. Kumarasamy College of Engineering, Karur 639113, Tamilnadu, India; 2 Department of Computer Science and Engineering, KGiSL Institute of Technology, Coimbatore 641035, Tamilnadu, India; nrk29@rediffmail.com; 3 Department of Information Technology, Karpagam College of Engineering, Coimbatore 641032, Tamilnadu, India; saravanakumaar2008@gmail.com; 4 Department of Electrical and Electronics Engineering, Dayananda Sagar College of Engineering, Bangalore 560078, Karnataka, India

**Keywords:** competitive learning, adaptive learning, multi-learning-based reptile search algorithm (MLBRSA), optimization, Q-learning, software requirement prioritization

## Abstract

In the realm of computational problem-solving, the search for efficient algorithms tailored for real-world engineering challenges and software requirement prioritization is relentless. This paper introduces the Multi-Learning-Based Reptile Search Algorithm (MLBRSA), a novel approach that synergistically integrates Q-learning, competitive learning, and adaptive learning techniques. The essence of multi-learning lies in harnessing the strengths of these individual learning paradigms to foster a more robust and versatile search mechanism. Q-learning brings the advantage of reinforcement learning, enabling the algorithm to make informed decisions based on past experiences. On the other hand, competitive learning introduces an element of competition, ensuring that the best solutions are continually evolving and adapting. Lastly, adaptive learning ensures the algorithm remains flexible, adjusting the traditional Reptile Search Algorithm (RSA) parameters. The application of the MLBRSA to numerical benchmarks and a few real-world engineering problems demonstrates its ability to find optimal solutions in complex problem spaces. Furthermore, when applied to the complicated task of software requirement prioritization, MLBRSA showcases its capability to rank requirements effectively, ensuring that critical software functionalities are addressed promptly. Based on the results obtained, the MLBRSA stands as evidence of the potential of multi-learning, offering a promising solution to engineering and software-centric challenges. Its adaptability, competitiveness, and experience-driven approach make it a valuable tool for researchers and practitioners.

## 1. Introduction

In the past few decades, there has been a noticeable increase in data dimensionality in real-world scenarios, resulting in a corresponding growth in the time and space complexity needed for their solution. The successful application of traditional mathematical optimization techniques frequently relies on the underlying symmetrical characteristics of the situation. While theoretical optimality guarantees exist for small-scale data-related issues, the practical application of these guarantees is challenging due to the significant time and space complexity involved [1,2]. Metaheuristic algorithms are commonly employed in the context of non-linear problems because of their advantageous characteristics, including straightforward principles, robustness against beginning values, and ease of implementation. In addition, it has been demonstrated that metaheuristic (MH) processes do not rely on the gradient of the fitness function, which has been shown to offer greater precision and practicality in terms of solution accuracy. Numerous MH algorithms have been presented since the onset of the 20th century. Moreover, MH techniques have been widely employed in diverse engineering domains, including but not limited to route planning, image processing, IoT task scheduling, software engineering job-shop scheduling, automatic control, mechanical engineering design, and power systems [3,4,5,6].

**Figure 1:**
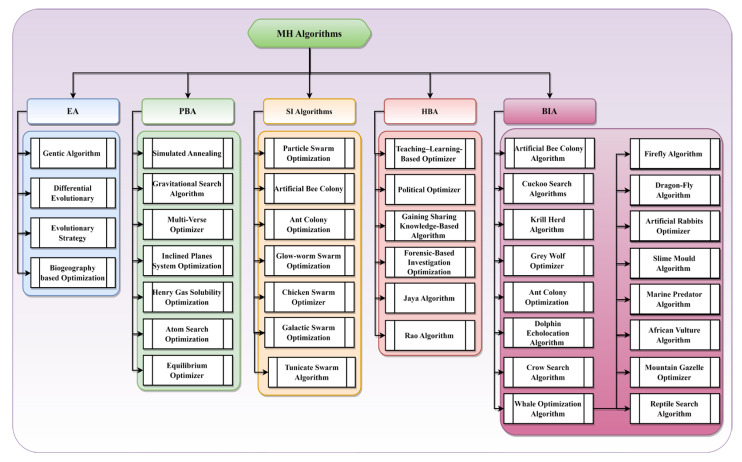
Classification of MH algorithms.

The increased pace of industrial expansion has led to a corresponding rise in the intricacy of optimization challenges that must be addressed. Numerous limited optimization problems exist that require urgent solution. These problems often exhibit numerous local optima within the feasible domain, rendering them inherently complex. Furthermore, the difficulty of addressing these problems is compounded when dealing with higher dimensions. The conventional approach to solving issues using classical derivatives is characterized by high processing costs, time requirements, and a tendency to converge towards local optima. These factors pose significant challenges in addressing the feasibility and economic considerations of actual situations. In contrast, heuristic algorithms encompass several approaches, such as greedy strategies and local search algorithms [7,8,9]. These algorithms rely on the inherent laws of the problem to obtain improved workable solutions. However, their effectiveness is highly contingent upon the problem being addressed, limiting their applicability and lacking generality. The proliferation of software for computers has led to the implementation and utilization of an increasing number of optimization methods. The MH algorithm is currently the most widely used optimization algorithm in the field. The MH optimization algorithms offer a cost-effective, straightforward, and efficient approach to addressing such difficulties. Optimal or near-optimal solutions can be obtained within a relatively brief timeframe [10,11,12]. The algorithm can identify the most effective approach for each problem instance and obtain the optimal solution. The MH algorithms are classified into two categories: non-nature-inspired and nature-inspired. The categorization of natural-inspired meta-heuristics encompasses four main groups: biologically inspired algorithms (BIA), physics-based algorithms (PBA), human-based algorithms (HBA), swarm intelligence (SI) algorithms, evolutionary algorithms (EA), and a miscellaneous category for those that do not fit into the groups mentioned above due to their diverse sources of inspiration, such as societal and emotional aspects [13,14]. Nature-based optimization approaches have experienced a process akin to the process of selection and elimination, resulting in their tendency to exhibit greater conciseness and superior performance compared to conventional techniques. The MH algorithms possess a straightforward structure, offer effortless operation, and exhibit a broad scope of applications, rendering them a highly favorable substitute for conventional methodologies [1,5]. The classification of MH algorithms is illustrated in Figure 1.

The SI algorithms are derived from the collective behavior exhibited by social insects, which has been developed over millions of years of evolutionary processes. Particle swarm optimization (PSO) is derived from the inherent characteristics of natural swarm particles [15]. The evolutionary algorithm is a probabilistic optimization technique that draws inspiration from the mechanisms of natural evolution. The genetic algorithm is derived from Darwinian theory [16]. PBA is predominantly obtained through the use of physical principles and chemical reactions. One example of an algorithm that draws inspiration from the behavior of systems with numerous degrees of freedom in thermal equilibrium at a finite temperature is simulated annealing (SA) [17]. A few other examples of PBAs are the gravitational search algorithm [18], Henry gas solubility optimization [19], equilibrium optimizer [20,21,22], and charged system search [23]. Human-based algorithms draw their inspiration mostly from human behavior. One illustrative instance is harmony search [24], which emulates the improvisational tactics employed by musicians. Several other widely used swarm intelligence algorithms include the krill–herd [25], artificial bee colony [26], cuckoo search algorithm [27], biogeography-based optimization [28], grey wolf optimizer (GWO) [29,30,31], whale optimization algorithm [32,33,34], dragon-fly algorithm [35], ant colony optimization [36], dolphin echolocation algorithm [37], firefly algorithm [38], slime mould algorithm [39,40,41], marine predator algorithm [42,43,44], mountain gazelle optimizer [45,46], African vulture algorithm [47], artificial rabbits optimizer [48], etc. The authors of [49] have used an improved sparrow search algorithm to estimate the parameters of the carbon fiber drawing process. The authors of [50] proposed an enhanced version of the snake optimizer for engineering design problems. The authors of [51] have proposed an improved whale optimization algorithm for cloud task scheduling problems. An improved version of the dragonfly algorithm with a neuro-fuzzy system has been proposed by [52] for wind speed forecasting.

These MH algorithms possess distinct attributes and are frequently employed in diverse computer science domains, including intrusion detection, parameter identification, path planning, engineering optimization, feature selection, fault diagnosis, text clustering problems, image segmentation, etc. Nevertheless, they continue to struggle with efficiently achieving a balance between the convergence rate and the accuracy of the solution. In broad terms, the optimization procedure of a MH algorithm comprises two distinct phases. The initial stage of the process involves exploration, wherein the algorithm thoroughly searches the feasible domain to identify the prospective region where the best solution could be found. The subsequent stage is characterized as exploitation, during which the algorithm conducts a more thorough search in pursuit of the ideal solution within a region that exhibits greater promise. These two phases exhibit a contradiction in their approach to addressing a problem, thus necessitating the development of an algorithm that can effectively navigate between exploration and exploitation. The algorithm must strike a judicious equilibrium to identify the best global solution without being trapped in a locally optimal one [53,54,55,56].

The no-free-lunch theorem demonstrates that algorithms do not universally apply to optimization issues [57]. Hence, it is crucial to enhance the efficiency of established algorithms. Numerous academics employ diverse methodologies to enhance pre-existing algorithms. For instance, the authors of [58] proposed incorporating an autonomous foraging mechanism called the remora optimization algorithm (ROA), which enables independent food discovery and less reliance on external sources. This integration significantly broadens the algorithm’s exploration capabilities and enhances its optimization accuracy. According to the authors of [59], incorporating roaming methods and lens opposition-based learning techniques enhanced the ability of the sand cat to conduct wide global searches. This integration also leads to accelerated convergence speed of the algorithm and successfully enhances its overall performance. Kahraman et al. (2020) introduced the fitness distance balance concept in their study [60]. Their fitness and distance values determine candidates’ scoring in the selection procedure. The population with the maximum score is chosen as the secondary solution, replacing the random individuals. This mechanism aims to increase the likelihood of an effective auxiliary solution, thereby improving algorithm efficiency and the likelihood of escaping local optima. In their study, the authors of [61] introduced the natural survivor method (NSM) as an alternative approach to solely relying on fitness values for evaluating and retaining individuals. To determine NSM scores, the researchers incorporated three parameters into their calculations. The factors mentioned above encompass the individual’s impact on the population, their influence on the mating pool, and their overall fitness worth. The scores of these three factors were dynamically weighted to decide the individual to retain by comparing their respective scores. According to the authors of [60,61,62], the potential for enhancing algorithm performance through effective measures exists. The data mentioned above clearly indicates that the enhanced MH algorithms have garnered significant interest within the realm of optimization.

The present study examines a new methodology known as the Reptile Search Algorithm (RSA), introduced by Abualigah et al. in 2021 [63]. The primary source of inspiration for this phenomenon is derived from the cooperative behavior exhibited by crocodiles during the act of predation. In their recent study, the authors of [64] introduced a novel approach called the hybrid RSA and ROA algorithm (RSAROA), which combines the use to optimize tasks and perform data clustering. This results in improved algorithm performance compared to other recently developed algorithms in particular problem domains. The authors of [65] introduced a modified version of the RSA specifically designed for numerical optimization problems. The utilization of the adaptive chaotic opposition-based learning strategy, shifting distribution estimation method, and elite alternative pooling technique effectively enhance the variety of the population, thereby achieving a balanced approach to exploration and exploitation. This ultimately leads to an improvement in the performance of the algorithm. The authors of [66] have introduced a new approach called the enhanced reptile search optimization algorithm using a chaos random drift and SA for feature selection. The RSA algorithm can be enhanced by including chaotic maps and SA techniques. This improved algorithm increased diversity within the initial population and improved algorithm progress. The authors of [67] have introduced a new approach called the improved RSA by the Salp swarm algorithm for medical image segmentation. This study aims to enhance the efficiency of the RSA by including the Salp swarm algorithm, with a specific focus on its application in the domain of image segmentation. This approach addresses the primary issues of early convergence and disparity in the search procedure as put forth by the original method.

One notable distinction between the RSA algorithm and other optimization algorithms is in the distinctive approach employed by the RSA to update the positions of search agents, which involves the utilization of four novel methodologies. For example, the behavior of surrounding prey is accomplished using two distinct locomotion methods: high-walking and belly-walking. Additionally, Crocodiles engage in communication and collaboration to effectively execute hunting strategies. The RSA aims to develop robust search algorithms that yield high-quality outcomes and generate novel solutions to address intricate real-world problems [68,69]. According to the authors, the RSA has effectively addressed artificial landscape functions and practical engineering challenges, surpassing other widely used optimization techniques [63]. The benchmark functions are mathematical functions commonly employed to assess the efficacy and efficiency of optimization techniques. Moreover, despite being classified as a stochastic population-based optimization method, the RSA has vulnerabilities in terms of maintaining population variety and avoiding local optima in the context of high-dimensional features. The factors mentioned above, as well as the distinguishing features of the RSA served as the impetus for undertaking this study to enhance its efficacy [70,71].

Q-Learning (QL) is a type of reinforcement learning (RL) technique that operates without the need for an explicit model of the environment. The integration of the QL and MH algorithm has been employed to enhance the optimization algorithm’s search capability, facilitated by advancements in RL [72,73,74,75]. The authors of the study conducted by [76] employed the RL technique to dynamically choose five strategies for enhancing the local search capabilities of the PSO. The authors of [77] have developed the differential evolutionary-QL (DEQL) method to produce a population of trials by utilizing QL. The QL determines the optimal choice of mutation and crossover techniques from a pool of four distinct strategies. The authors in the study by [78] employed a combination of PSO and RL techniques to develop individual Q-tables for each particle. Additionally, they implemented a dynamic selection mechanism for adjusting the particle characteristics. The authors of [79] introduced the QL-embedded sine-cosine algorithm (Q-SCA) as a means of parameter control. Using the QL technique can potentially expedite the escape of the sine cosine algorithm from local optima. The authors of [80] propose that the exploration ability of the QL algorithm can be improved by dynamically selecting the search strategy of the arithmetic optimization algorithm (AOA). In the literature previously mentioned, the QL algorithm was employed to optimize the approach for a certain algorithm. The presence of certain limits may impede the resolution of various optimization challenges. The QL algorithm employs a reward function assigned a constant value, hence creating a situation perceived as unjust for persons who have made more advancements. Furthermore, generating a Q-table for each individual results in a significant increase in spatial complexity. In order to tackle this issue, it is possible to devise a hybrid approach incorporating a RL algorithm. This approach aims to optimize the selection of a meta-heuristic algorithm, hence maximizing the benefits achieved at various phases [77,79]. It has been observed that the aforementioned literature shares a common objective, namely, to mitigate algorithmic precocity and achieve a harmonious equilibrium between exploration and progress. To address the concerns above, the following measures have been undertaken. A QL mechanism can enhance crocodiles’ spatial exploration and exploitation capabilities. In addition, competitive learning and adaptive learning mechanisms can be used to further improve the performance of the RSA. Hence, this study presents a new approach, namely the Multi-Learning-Based Reptile Search Algorithm (MLBRSA), for addressing the global optimization and software requirement prioritization (SRP) problems. The choice of appropriate methods for boosting and enhancement frameworks should be guided by the algorithm’s challenges and the characteristics of the optimization issues it aims to solve. QL, competitive learning, and adaptive learning were selected due to their direct relevance in addressing several inherent problems associated with RSA, including the convergence towards local optima, sensitivity to parameters, and the absence of a balanced exploration-exploitation trade-off. Alternative frameworks may potentially create superfluous intricacy or may not fit as well with RSA’s particular dynamics and objectives. The following are the major contributions of this study:

- The multi-learning approach is proposed to improve the performance of the RSA;
- Dynamic learning with more rewards in different situations increases the diversity of solutions in the population and the robustness of RSA;
- Validated using 23 benchmark test functions with different dimensions and five constrained engineering design problems;
- Validated using the software requirement prioritization optimization problem;
- Compared with state-of-the-art algorithms, including the original RSA.

The paper is organized as follows: Section 2 briefly discusses the concepts of the original RSA; Section 3 comprehensively presents the proposed MLBRSA; Section 4 details the SRP problem, and the objective function and constraints are also discussed; Section 5 discusses the results of the 23 benchmark functions with different dimensions and five engineering optimization problems; Section 5 also discusses the results obtained for the SRP problem; and Section 6 concludes the paper.

## 2. Reptile Search Algorithm

The RSA method, created by Abualigah et al. [69], is an innovative optimization technique that emulates the encircling and hunting behaviors of crocodiles. This section elucidates the exploration and exploitation skills of the RSA, which are derived from its intelligent surroundings and hunting strategies employed to capture prey. The RSA is a population-based approach and does not rely on gradient information. It can address intricate and straightforward optimization issues while adhering to predefined limitations.

### 2.1. Initialization

The initial candidate solutions are constructed randomly during this stage, as described in Equation (1).

(1) $$\begin{array}{c} X=\left[\begin{array}{ccccc} x_{1,1} & \ldots & x_{1,j} & x_{1,n-1} & x_{1,n}\\ x_{2,1} & \ldots & x_{2,j} & x_{2,n-1} & x_{2,n}\\ \ldots & \ldots & x_{i,j} & x_{i,n-1} & x_{i,n}\\ \vdots & \vdots & \vdots & \vdots & \\ x_{N-1,1} & \ldots & x_{N-1,j} & \ldots & x_{N-1,n}\\ x_{N,1} & \ldots & x_{N,j} & x_{N-1,n} & x_{N,n} \end{array}\right] \end{array}\;$$

where X denotes the candidate solutions and $x_{ij}$ denotes the jth position of the ith solution, N  denotes the population size, and n denotes the problem dimension.

(2) $$\begin{array}{c} x_{ij}=rand(UB-LB)+LB,j=1,2,\ldots ,n \end{array},\;i=1,2,...,N\;$$

where UB and LB signify the upper and lower bounds, and rand denotes the random number between [0, 1].

### 2.2. Exploration Phase

Crocodiles employ two distinct techniques, namely high walking and belly walking, throughout their encircling procedure. The RSA incorporates a balanced approach between exploration and exploitation, which can be likened to encircling and hunting, respectively. This approach is guided by four conditions, which involve dividing the entire number of iterations into four distinct portions. The exploration processes employed in RSA primarily focus on two prominent search strategies, namely high walking and belly walking, which are utilized to navigate the search space and identify optimal solutions. The high walk strategy is characterized by the condition $t\leq \frac{T}{4}$. The belly walk motion strategy is characterized by conditions $t\leq 2\frac{T}{4}$ and $t>\frac{T}{4}$. This implies that the condition is satisfied for approximately half of the exploration iterations conducted during the high walk, while the remaining half is satisfied during the belly walk. The formula for updating the position is stated in Equation (3) during the exploration phase.

(3) $$x_{\left(i,j\right)}\left(t+1\right)=\left\{\begin{array}{c} {Best}_{j}\left(t\right)-\eta_{\left(i,j\right)}\left(t\right)\times \beta -R_{\left(i,j\right)}\left(t\right)\times rand,t\leq \frac{T}{4}\\ {Best}_{j}\left(t\right)\times x_{r1,j}\times ES\left(t\right)\times rand,\;t\leq 2\frac{T}{4}\;\mathrm{and}\;t>\frac{T}{4}\; \end{array}\right.\;$$

(4) $$\eta_{\left(i,j\right)}={Best}_{j}\left(t\right)\times P_{\left(i,j\right)}\;$$

(5) $$R_{\left(i,j\right)}=\frac{Best_{j}\left(t\right)-x_{\left(r_{2},j\right)}}{Best_{j}\left(t\right)+\epsilon }\;$$

(6) $$ES(t)=2\times r_{3}\times \left(1-\frac{1}{T}\right)$$

(7) $$\begin{array}{c} P_{\left(i,j\right)}=\alpha +\frac{x_{\left(i,j\right)}\;-\;M\left(x_{i}\right)}{Best_{j}\left(t\right)\times \left({UB}_{\left(j\right)}-{LB}_{\left(j\right)}\right)+\epsilon } \end{array}\;$$

(8) $$\begin{array}{c} M\left(x_{i}\right)=\frac{1}{n}\sum_{j=1}^{n}x_{\left(i,j\right)} \end{array}\;$$

where ${Best}_{j}\left(t\right)$ signifies the best solution found so far, rand signifies the uniform random number in the range of 0 and 1, T denotes the maximum iterations, t denotes the current iteration, β denotes the control parameter guides the exploration, and its value is 0.1, $\eta_{\left(i,j\right)}$ denotes the operator who controls the exploration, $R_{\left(i,j\right)}$ denotes the factors that reduce the search area, ϵ denotes the epsilon (floating-point relative accuracy and is equal to 2.2204 × 10^−16^), $x_{r1,j}$ and $x_{r2,j}$ denotes the random population positions of the ith solution, $ES\left(t\right)$ denotes the random factors between [−2, 2], $r_{3}$ denotes the random integer between [−1, 1], $P_{\left(i,j\right)}$ denotes the difference between the current solution and the best solution obtained so far, α is constant, which drives the exploration, and its value is 0.1, and $M\left(x_{i}\right)$ denotes the mean position of the ith solution.

### 2.3. Exploitation Phase

Crocodiles employ two distinct methods, namely collaboration and coordination, throughout their hunting attempts. The approaches employed in this study imitate the exploitation search formulated according to Equation (9). The hunting coordination approach in this phase is determined by the criteria $t\leq 3\frac{T}{4}$ and $t>2\frac{T}{4}$; otherwise, the hunting collaboration approach is implemented. The position update equation for the exploitation in the initial RSA is described in Equation (9).

(9) $$x_{\left(i,j\right)}\left(t+1\right)=\left\{\begin{array}{c} {Best}_{j}\left(t\right)\times P_{\left(i,j\right)}\left(t\right)\times rand,t>\frac{T}{2}\mathrm{and}\;t\leq 3\frac{T}{4}\\ {Best}_{j}\left(t\right)-\eta_{\left(i,j\right)}\left(t\right)\times \epsilon -R_{\left(i,j\right)}\left(t\right)\times rand,t>3\frac{T}{4}\mathrm{and}\;t\leq T\; \end{array}\right.\;$$

where ${Best}_{j}\left(t\right)$ denotes the best solution found so far, $\eta_{\left(i,j\right)}\left(t\right)$ denotes the hunting variable computed using Equation (4), rand means the random number between 0 and 1, $R_{\left(i,j\right)}\left(t\right)$ is computed using Equation (5), and $P_{\left(i,j\right)}\left(t\right)$ computed using Equation (7). Ultimately, in the unlikely scenario that the proposed candidate’s location is nearer to the sustenance source than the current candidate, the reptile continues to relocate to the new candidate’s location and commences the subsequent iteration. The pseudocode of the original RSA is shown in Algorithm 1.
**Algorithm 1**: Pseudocode of the Reptile Search AlgorithmInitialize the population size, maximum number iterations, ϵ, α and β. Initialize the population position randomly and their respective solution.**While** t<T  Calculate the fitness, find the best solution and update ES  using Equation (6).
   **For**
 i=1: N

    **For**
 j=1: n
      Update $\eta_{\left(i,j\right)}$$,\;R_{\left(i,j\right)}$$,\;P_{\left(i,j\right)}$$,\;\mathrm{and}\;M\left(x_{i}\right)$ using Equations (4), (5), (7) and (8).      **If**
$\;t\leq \frac{T}{4}$
           Update using Equation (3a).
      **else if**
$\;t\leq 2\frac{T}{4}and\;t>\frac{T}{4}$
           Update using Equation (3b).
      **else if**
$\;t>\frac{T}{2}and\;t\leq 3\frac{T}{4}$
           Update using Equation (9b).
      **else**
         Update using Equation (9b).
      **End if**

    **End for**

  **End for**
**End while** **Return**: Best position and the respective solution

## 3. Proposed Multi-Learning-Based Reptile Search Algorithm

The RSA is a nature-inspired metaheuristic algorithm that mimics the hunting behavior of reptiles. Like many metaheuristic algorithms, the RSA has its strengths but has certain limitations or defects. Some potential defects of the original RSA include: (i) the RSA, like many optimization algorithms, can sometimes get trapped in local optima, especially in complex search spaces with multiple peaks and valleys. This means the algorithm might converge to a sub-optimal solution rather than the global optimum, (ii) the performance of the RSA can be sensitive to its parameter settings, such as the values of α and β, (iii) when dealing with high-dimensional problems, the RSA might exhibit slow convergence rates, (iv) the original RSA might not always strike the right balance between exploration and exploitation, (v) there might be situations where the algorithm becomes stagnant, with solutions oscillating around certain values without significant improvements, (vi) the computational cost can increase significantly, and the algorithm might struggle to find good solutions within a reasonable time frame, and (vii) the original RSA does not have mechanisms to adapt its parameters or strategies based on the problem’s characteristics or its current performance [68,69,70,71]. This lack of adaptability can hinder its performance on diverse problems. Therefore, it is essential to note that while the RSA has these potential defects, it also has strengths, and its performance can be problem-dependent. The proposed enhancements, including the integration of Q-learning, competitive learning, and adaptive learning, aim to address some defects and improve the algorithm’s robustness and efficiency [81,82].

### 3.1. Reinforcement Learning

**Figure 2:**
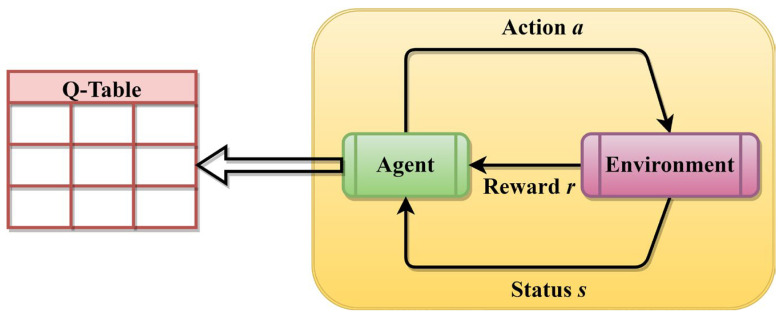
The framework of the RL.

In history, numerous noteworthy advancements have emerged in the field of reinforcement learning. This area of study can be classified into two distinct categories: policy-based approaches and value-based methods. The Q-learning algorithm is commonly regarded as a representative example of value-based techniques. During the process of learning, the agent engages in actions that have the highest predicted Q-values in order to compute the optimum course of action. The objective is to establish a reciprocal relationship with the surrounding environment utilizing the agent, afterwards acquiring the highest possible reward to attain the most advantageous course of action, as seen in Figure 2. The Q-learning comprises state-space $S=\{s_{1},s_{2},\cdots ,s_{m}\}$, action space $A=\{a_{1},a_{2},\cdots ,a_{n}\}$, an environment, the learning agent, and the reward function R. The Q-table undergoes dynamic updates dependent on the reward, and its computation is performed as follows [78,79,82]:

(10) $$Q\left(s_{t+1},a_{t+1}\right)=Q\left(s_{t},a_{t}\right)+\lambda \left[r_{t+1}+\gamma \underset{a}{max}Q\left(s_{t+1},a\right)-Q\left(s_{t},a_{t}\right)\right]\;$$

where $a_{t}$ denotes the current action, $s_{t+1}$ denotes the next state, $s_{t}$ denotes the current state, $r_{t+1}$ denotes the instant reinforcement reward learned from the accomplishment of $a_{t}$ at $s_{t}$, λ denotes the learning rate, γ denotes the discount factor, and $Q\left(s_{t+1},a\right)$ denotes the predicted Q-value when a performed at $s_{t+1}$.

The Q-table can be represented as a m×n matrix where n and m denote the number of actions and states correspondingly. The Q-table can be described as a mapping table that associates the current state of execution with certain actions and their corresponding future rewards. The pseudocode of the QL is presented in Algorithm 2.
**Algorithm 2:** Pseudocode of the QL AlgorithmInitialize the states s and the action a.**For**$\mathrm{each}\;s_{i}$ and $a_{i}$
$\;\;\;\mathrm{Set}\;Q\left(s_{i},a_{i}\right)=0$.**End For**Choose the initial state s randomly.**While** the criteria not reached   Select the best action from the current state from the Q-table.   Execute the action and then get the immediate reward.$\;\;\;\mathrm{Determine}\;\mathrm{the}\;\mathrm{new}\;\mathrm{state}\;s_{t+1}$.   Obtain the respective maximum Q-value.    Update the Q-table using Equation (10) and update the state.**End While**

### 3.2. Competitive Learning

When applied to optimization algorithms like the RSA, competitive learning determines which solutions (or “reptiles” in the context of the RSA) perform best and should influence or guide the search process. In the proposed MLBRSA, solutions compete based on their fitness values. The solution with the best (e.g., lowest) fitness value “wins” the competition. The winning solution influences the position updates of other solutions. This is done to guide the search towards promising regions of the search space. Competitive learning introduces a form of guided exploration. While random exploration helps search the entire solution space, the influence of the best solution ensures that the search is also exploitative, focusing on areas that have yielded good solutions. As the search progresses and different solutions become winners in different iterations, the search direction and focus can dynamically change, allowing the algorithm to adapt to complex landscapes [83,84,85]. In this study, competitive learning influences how solutions are updated. The winning solution (the one with the best fitness) provides a reference or guide for updating other solutions [81]. This ensures that (i) the search is biased towards regions of the search space that have yielded good solutions, (ii) solutions can escape local optima by being influenced by the global best or other high-performing solutions, and (iii) the diversity of solutions is maintained, as not all solutions are pulled towards the best one, but are updated with a mix of exploration and exploitation.

**Figure 3:**
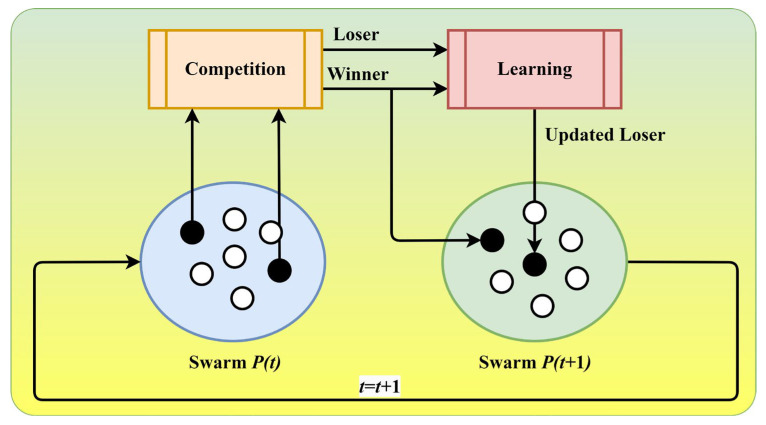
The framework of competitive learning.

In each successive iteration, reptiles are chosen randomly in pairs from the existing population to engage in competitive interactions. Following each competition, the participant with a lower fitness value, referred to as the loser, undergoes an update process by assimilating knowledge from the winner. Conversely, the winner is to be directly included in the population of the subsequent iteration. The framework of competitive learning is provided in Figure 3. The first step in competitive learning is the winner selection. For the given set of solutions X with the fitness F, the winning solution $x_{winner}\;$ is the one with the best fitness:

(11) $$x_{winner}=arg\underset{x\in X}{\;\min}F\left(x\right)$$

The update of a solution $x_{i}$ considering the winning solution $x_{winner}$ can be modeled as follows:

(12) $$x_{i}^{new}=x_{i}+\mu \times \left(x_{winner}-x_{i}\right)+other\;terms$$

where μ is a factor determining the extent of influence and other terms, represent other update components (e.g., random exploration). In this study, the value of μ is selected as 0.1, i.e., 10% of the solutions are moving towards the winning solution. In competitive learning, the influence factor, or learning rate, critically shapes the RSA’s behavior. A higher rate, exemplified by 50%, accelerates adaptation to input data, fostering quicker convergence. However, this swiftness can lead to overshooting and instability, potentially delaying generality. Conversely, a lower rate, like 1%, ensures a more stable learning process but may sacrifice speed, potentially causing delays in convergence and adaptation. In order to strike a balance, a 10% learning rate often proves optimal, offering a moderate convergence speed without compromising stability excessively. This choice is typically justified through empirical validation, where the learning rate’s impact determines the most effective compromise between convergence speed and stability. Tailoring the learning rate to the problem’s specific characteristics and considering computational resources ensures an informed and efficient choice in the competitive learning process. The pseudocode of competitive learning is provided in Algorithm 3.
**Algorithm 3:** Pseudocode of the Competitive LearningInitialize solutions X randomly. Evaluate the fitness of each solution in X.**While** not converged:  $\mathrm{Determine}\;x_{winner}$, the solution with the best fitness in X.  **For** each solution $x_{i}$ in X:$\;\;\;\;\mathrm{Calculate}\;\mathrm{the}\;\mathrm{competitive}\;\mathrm{influence:}\;influence=\mu \times (x_{winner}-x_{i})$.$\;\;\;\;\mathrm{Update}\;x_{i}$$\;\mathrm{considering}\;\mathrm{the}\;\mathrm{influence}\;\mathrm{and}\;\mathrm{other}\;\mathrm{factors}:\;x_{i}=x_{i}+influence+otherupdatedterms$.$\;\;\;\;\mathrm{Ensure}\;x_{i}$$\;\mathrm{is}\;\mathrm{within}\;\mathrm{bounds}\;\mathrm{and}\;\mathrm{evaluates}\;\mathrm{the}\;\mathrm{fitness}\;\mathrm{of}\;x_{i}$.
  **End For**
**End While**

In this study, competitive learning provides a mechanism to guide the search using the best-found solutions. This balance between exploration and exploitation can enhance the algorithm’s performance in finding optimal or near-optimal solutions.

### 3.3. Adaptive Learning

Adaptive learning refers to the ability of an algorithm to adjust its parameters or behavior based on its performance or the characteristics of the problem being solved. Adaptive learning can be crucial for balancing exploration and exploitation in optimization algorithms. Adaptive learning often involves dynamically adjusting algorithm parameters, such as learning rates, based on the algorithm’s performance. The algorithm uses feedback, typically in solution quality or convergence speed, to decide how to adjust its parameters. In order to adapt to the problem’s landscape, the algorithm can converge faster to high-quality solutions. Adaptive mechanisms can help the algorithm escape local optima by adjusting its search behavior [86].

In the proposed MLBRSA, adaptive learning influences how the algorithm updates its solutions. Specifically, (i) parameters like α and β in the MLBRSA are adjusted based on the best solution performance. If the best solution improves, the parameters might be increased to intensify the search around it. If the best solution stagnates, the parameters might be decreased to diversify the search, and (ii) by adjusting parameters like α and β, the algorithm can dynamically shift between exploration and exploitation, ensuring a good balance throughout the search process. The feedback $F_{feedback}$ can be calculated as the difference in the best solution’s fitness between two consecutive iterations as follows:

(13) $$F_{feedback}=F_{best}\left(t\right)-F_{best}\left(t-1\right)$$

where $F_{best}\left(t\right)$ denotes the current best solution and $F_{best}\left(t-1\right)$ denotes the previous best solution. Equation (14) is used to update the parameters adaptively. The term P represents the parameters to be adapted during the iterative procedure, i.e., α and β, in this study. The update of a parameter P based on the feedback can be modeled as follows:

(14) $$\left\{\begin{array}{c} P\left(t\right)+\delta_{increase},\;if\;F_{feedback}>0\\ P\left(t\right)-\delta_{decrease},\;if\;F_{feedback}\leq 0 \end{array}\right.\;$$

where $\delta_{increase}$ and $\delta_{decrease}$ denote small positive constants determining the magnitude of the parameter adjustment. The pseudocode of adaptive learning is provided in Algorithm 4.
**Algorithm 4:** Pseudocode of the Adaptive LearningInitialize solutions X randomly. Evaluate the fitness of each solution and initialize α and β and $F_{best}\left(t-1\right)=$ infinity.**While** not converged:    $\mathrm{Determine}\;F_{best}\left(t\right)$, the best fitness in X.    **For**$\;\mathrm{each}\;\mathrm{solution}\;x_{i}$ in X:$\;\;\;\;\;\mathrm{Update}\;x_{i}$ using current parameters (α and β). $\;\;\;\;\;\mathrm{Ensure}\;x_{i}$$\;\mathrm{is}\;\mathrm{within}\;\mathrm{bounds}\;\mathrm{and}\;\mathrm{evaluates}\;\mathrm{the}\;\mathrm{fitness}\;\mathrm{of}\;x_{i}$.                    **End For**
                **//Adaptive Learning//**    Find the feedback value by $F_{feedback}=F_{best}\left(t\right)-F_{best}\left(t-1\right)$.    **If**$\;F_{feedback}>0$:$\;\;\;\;\;\mathrm{Increase}\;\mathrm{parameters}\;(\alpha +=\delta_{increase}$ and $\beta +=\delta_{increase}$).
      **Else**:
$\;\;\;\;\;\mathrm{Decrease}\;\mathrm{parameters}\;(\alpha -=\delta_{decrease}$ and $\beta -=\delta_{decrease}$).
      **End If**
$\;\;F_{best}\left(t-1\right)=F_{best}\left(t\right)$**End While**

In the proposed MLBRSA, adaptive learning provides a mechanism to adjust the algorithm’s behavior based on performance. This dynamic adjustment can help the algorithm respond better to the challenges of the problem’s landscape, enhancing its ability to find optimal or near-optimal solutions.

### 3.4. Multi-Learning Reptile Search Algorithm

This subsection explains the step-by-step procedure of the proposed MLBRSA. The following steps describe the formulation of the proposed algorithm.

**Step 1—Initialize MLBRSA:** The algorithm initializes the MLBRSA. This involves setting up the initial population of solutions and defining the search space boundaries. Given a population size N, dimension n, and search space boundaries LB and UB, initialize the population X:

(15) $$\begin{array}{c} x_{ij}=rand(UB-LB)+LB,j=1,2,\ldots ,n \end{array},\;i=1,2,...,N\;$$

**Step 2—QL Decision:** At this step, the algorithm uses QL to decide the next action for each solution. This decision is based on past experiences and the expected reward of taking a particular action in the current state. For each solution $x_{i}$, decide the next action based on the Q-table Q and an exploration rate ξ:

(16) $${Actions}_{i}=\left\{\begin{array}{c} randi\left(\left[1,4\right]\right),\;if\;rand\left(0,1\right)<\xi \;\\ {argmax}_{a}Q\left(i,a\right),\;Otherwise \end{array}\right.$$

**Step 3—Competitive Learning:** Here, the solutions compete against each other based on their fitness values. Only the best solutions (winners) can update their positions, while the others remain unchanged. This introduces a survival-of-the-fittest dynamic. For the given set of solutions X with the fitness F, the winning solution $x_{winner}\;$ is the one with the best fitness:

(17) $$x_{winner}={argmin}_{i}F\left(x_{i}\right)\;$$

The competition influence is calculated as follows:

(18) $$influence=\mu \times \left(x_{winner}-x_{i}\right)$$

where μ is the influence factor and its value is 0.1, i.e., 10%.

**Step 4—Adaptive Learning:** The algorithm evaluates its performance and dynamically adjusts its parameters (α and β). This self-tuning mechanism ensures that the algorithm remains flexible and adaptable to the problem’s characteristics, dynamically adjusting the parameters α and β based on the performance difference ∆F between two consecutive iterations:

(19) $$∆F=F_{best}\left(t\right)-F_{best}\left(t-1\right)$$

The dynamic parameters are as follows:

(20) $$\alpha =\left\{\begin{array}{c} \alpha +\delta ,\;if\;∆F<0\\ \alpha -\delta ,\;\mathrm{Otherwise} \end{array}\right.$$

(21) $$\beta =\left\{\begin{array}{c} \beta +\delta^{\prime },\;if\;∆F<0\\ \beta -\delta^{\prime },\;\mathrm{Otherwise} \end{array}\right.$$

where δ and $\delta^{\prime }$ are small positive constants, and their value is 0.01 and 0.001, respectively. 

**Step 5—Update & Iterate:** Based on the decisions from the previous steps, the algorithm updates the positions of the solutions. It then checks for convergence criteria. If the criteria are not met, the algorithm returns to the QL step and iterates until the end conditions are satisfied. Update the position of each solution based on the selected action using Equation (22):

(22) $$x_{\left(i,j\right)}\left(t+1\right)=\left\{\begin{array}{c} \begin{array}{c} {Best}_{j}\left(t\right)-\eta_{\left(i,j\right)}\left(t\right)\times \beta -R_{\left(i,j\right)}\left(t\right)\times rand+influence,\;\mathrm{if}\;\mathrm{Action}=1\\ {Best}_{j}\left(t\right)\times x_{r1,j}\times ES\left(t\right)\times rand+influence,\;\mathrm{if}\;\mathrm{Action}=2\; \end{array}\\ {Best}_{j}\left(t\right)\times P_{\left(i,j\right)}\left(t\right)\times rand+influence,\;\mathrm{if}\;\mathrm{Action}=3\\ {Best}_{j}\left(t\right)-\eta_{\left(i,j\right)}\left(t\right)\times \epsilon -R_{\left(i,j\right)}\left(t\right)\times rand+influence,\;\mathrm{if}\;\mathrm{Action}=4\; \end{array}\right.$$

**Step 6**—Iterate until a stopping criterion is met and return the best solution.

The pseudocode of the suggested MLBRSA is presented in Algorithm 5.
**Algorithm 5:** Pseudocode of the Proposed MLBRSAInitialize solutions X randomly. Evaluate the fitness of each solution and Initialize α and $\beta \;and\;F_{best}\left(t-1\right)=\;$ infinity. Initialize the states s and the action a.**For**$\;\mathrm{each}\;s_{i}$ and $a_{i}$
$\;\;\mathrm{Set}\;Q\left(s_{i},a_{i}\right)=0$.**End For**Choose the initial state s randomly.**While** not converged:    Determine $x_{winner}$, the solution with the best fitness in X.    Determine $F_{best}\left(t\right)$, the best fitness in X.    **For** each solution $x_{i}$ in X:        QL Action Selection (Random or greedy action).$\;\;\;\;\;\;\;\mathrm{Calculate}\;\mathrm{the}\;\mathrm{competitive}\;\mathrm{influence}:\;influence=\mu \times (x_{winner}-x_{i})$.        Select the best action from the current state from the Q-table.        Execute the action and then get the immediate reward.        Decide action using QL. If rand(0,1)<ε, choose a random action; else, use Q-table.$\;\;\;\;\;\;\;\mathrm{Update}\;x_{i}$ considering the influence and other factors using Equation (22).$\;\;\;\;\;\;\;\mathrm{Determine}\;\mathrm{the}\;\mathrm{new}\;\mathrm{state}\;s_{t+1}$.        Obtain the respective maximum Q-value.         Update the Q-table using Equation (10) and update the state.$\;\;\;\;\;\;\;\mathrm{Ensure}\;x_{i}$$\;\mathrm{is}\;\mathrm{within}\;\mathrm{bounds}\;\mathrm{and}\;\mathrm{evaluates}\;\mathrm{the}\;\mathrm{fitness}\;\mathrm{of}\;x_{i}$.    **End For**
    **For**$\;\mathrm{each}\;\mathrm{solution}\;x_{i}$ in X:$\;\;\;\;\;\;\mathrm{Update}\;x_{i}$ using current parameters (α and β). $\;\;\;\;\;\;\mathrm{Ensure}\;x_{i}$$\;\mathrm{is}\;\mathrm{within}\;\mathrm{bounds}\;\mathrm{and}\;\mathrm{evaluates}\;\mathrm{the}\;\mathrm{fitness}\;\mathrm{of}\;x_{i}$.
    **End For**
    Find the feedback value by $\;∆F=F_{best}\left(t\right)-F_{best}\left(t-1\right)$.    **If**${\;F}_{feedback}>0$:$\;\;\;\;\;\;\mathrm{Increase}\;\mathrm{parameters}\;(\alpha +=\delta_{increase}$ and $\beta +=\delta_{increase}$).    **Else**:$\;\;\;\;\;\;\mathrm{Decrease}\;\mathrm{parameters}\;(\alpha -=\delta_{decrease}$ and $\beta -=\delta_{decrease}$).
    **End If**
    $F_{best}\left(t-1\right)=F_{best}\left(t\right)$
**End While****Return:** Best solution.

### 3.5. Computational Complexity

Analyzing the time and space complexity of the MLBRSA can be more nuanced than traditional algorithms due to their stochastic nature and dependence on parameters. The time complexity is provided as follows: (i) The initialization of (N) solutions with (n) dimensions takes $\left(O\left(N\times n\right)\right)$; (ii) QL Decision: for each of the (N) solutions, deciding the next action based on the Q-table is $\left(O\left(1\right)\right)$, so in total, it is $\left(O\left(N\right)\right)$; (iii) Competitive Learning: Finding the best solution based on fitness evaluation is $\left(O\left(N\right)\right)$; (iv) Adaptive Learning: Adjusting parameters based on performance is $\left(O\left(1\right)\right)$ for each solution, so $\left(O\left(N\right)\right)$ in total; and (v) Update & Iterate: Updating the position of each solution and checking for convergence for each of the (T) iterations is $(O\left(N\times n\right)).$ Therefore, the overall time complexity for the algorithm for (T) iterations is: $\left[O\left(T\times \left(N\times n+N+N+N\right)\right)=O\left(T\times N\times n\right)\right]$. The space complexity is provided as follows: (i) Population Matrix: Storing (N) solutions, each with (n) dimensions, requires $\left(O\left(N\times n\right)\right)$ space; (ii) Q-table: Assume a discrete state and action space for QL; the Q-table’s size would be $\left(O\left(\mathrm{states}\times \mathrm{actions}\right)\right)$. However, in the MLBRSA, this might be abstracted or approximated so that the exact space complexity can vary based on the implementation; and (iii) Auxiliary Variables: Variables like (α), (β), fitness values, etc., would take $\left(O\left(N\right)\right)$ space. Therefore, the overall space complexity is: $\left[O\left(N\times n+\mathrm{states}\times \mathrm{actions}+N\right)\right]$.

## 4. Software Requirements Prioritization (SRP) Problem

Software Requirements Prioritization (SRP) optimization problem addresses the challenge of ranking software requirements in order of importance. In the realm of software development, it is crucial to determine which features or functionalities should be developed first, considering constraints like time, budget, and resources. SRP ensures that the most critical requirements, which offer maximum value to stakeholders and end-users, are addressed promptly. Traditional methods often fall short in this multi-faceted decision-making process. Hence, optimization techniques are employed in SRP to evaluate and prioritize requirements holistically, ensuring that software products are both high-quality and aligned with user needs and business objectives.

### 4.1. Introduction

Over the past several decades, the technological landscape has witnessed significant advancements, leading to the emergence of intricate and sophisticated software systems. Given their heightened sensitivity to various factors, developing these large-scale software systems is a delicate process. Creating a comprehensive and quality software system involves input from multiple stakeholders. These stakeholders play a pivotal role in outlining the essential features, functionalities, and capabilities that the software must encompass. Their collective vision and expectations lay the foundation for the software’s overall quality and performance. Central to the development of high-quality software is the process of requirements engineering. This process is the backbone of software development, ensuring the system is built on a solid foundation of well-defined and well-understood requirements. Even though these requirements form the basis for creating a highly adaptable system, their importance cannot be overstated. The journey of requirements engineering is multi-faceted, comprising several critical phases. These include (i) Elicitation: This is the initial phase where the requirements are gathered from various sources, primarily stakeholders; (ii) Analysis: Here, the gathered requirements are scrutinized to ensure clarity and feasibility; (iii) Documentation: This phase involves recording the analyzed requirements in a structured manner; (iv) Verification: This ensures that the documented requirements align with the stakeholders’ expectations; (v) Validation: This phase checks the feasibility and relevance of the requirements in the context of the software’s objectives; and (vi) Prioritization: In this phase, requirements are ranked based on their significance and impact on the software’s overall functionality [87,88,89].

The prioritization process is particularly crucial. It ensures that the software system is developed in a structured manner, allowing for the timely creation of its major components. This not only guarantees the software’s quality but also takes into account various other considerations that might influence its development and deployment. The genesis of any software development process lies in accurately identifying and understanding its requirements. Even minor oversights in this phase can have cascading effects, leading to inflated cost projections, extended development durations, compromised quality, reduced client satisfaction, and, in extreme cases, the complete failure of the project. Specific elicitation techniques ensure that the client’s requirements are accurately captured. These techniques aim to gather software requirements directly from the stakeholders, ensuring that the software meets their expectations and needs [90,91]. During the elicitation phase, requirements are broadly categorized into two types: (i) Functional Requirements: These pertain to the specific functions and features the software should possess, and (ii) Non-Functional Requirements: These are criteria against which the functional requirements are evaluated, ensuring that the software meets certain quality standards. As the software moves into the implementation phase, these requirements are prioritized based on their importance. This ranking ensures that the most critical components are addressed first, paving the way for a systematic and efficient development process [92,93].

It is crucial to swiftly identify and address customer needs, ensuring their utmost satisfaction. Certain software approaches, like agile’s incremental development, involve multiple releases, each with its distinct set of requirements. Given the myriad technical challenges and conflicts developers encounter during product development, selecting a subset of requirements is vital to maximizing customer satisfaction. However, choosing the best subset from various requirements is challenging. In order to aid with decision-making, it is essential to introduce a methodology that pinpoints the most optimal subset of requirements. Successful requirement analysis hinges on ranking software requirements based on quality, cost, delivery time, and available resources. In software requirement prioritization (SRP), stakeholders are pivotal in prioritizing requirements. Their analysis is crucial, especially since different stakeholders might perceive the same requirement differently. This variance in perception can be particularly pronounced between seasoned professionals and newcomers. Linguistic terms are employed to articulate requirement preferences. Additionally, fuzzy numbers are utilized to quantify ambiguous and subjective data [94,95,96].

Customer satisfaction and precise requirements identification are paramount when formulating an optimal subset for the Next Release Problem (NRP-hard). The NRP-hard refers to the challenge of determining which features or requirements should be included in the next version of a software product, taking into account various constraints and objectives. Traditional optimization methods discussed in Section 1, which often focus on a singular objective or criterion, have proven inadequate in addressing the multi-faceted nature of the NRP-hard. These conventional methods, being linear and singular in their approach, often miss out on capturing the intricate interplay of various factors that influence the decision-making process for the next release. The complexity of the NRP-hard arises from balancing multiple objectives, such as cost, time, resource allocation, and, most importantly, customer satisfaction. Since each requirement might have different implications for these objectives, finding an optimal subset is not straightforward. For instance, while customers might highly desire one requirement, it might also be resource-intensive, pushing the release date further. Therefore, relying solely on traditional optimization methods can lead to suboptimal decisions. These methods might overlook certain critical requirements or prioritize less impactful ones, ultimately failing to deliver a product version that truly resonates with customer needs and organizational goals. In essence, to effectively tackle the NRP-hard, there is a need for more reliable optimization techniques that can consider and balance the myriad of factors and constraints involved [97,98,99]. Therefore, in this study, the proposed MLBRSA is applied to the SRP problem to handle the NRP-hard optimization problem. The performance of the MLBRSA is compared with other algorithms to prove its superiority.

### 4.2. Problem Formulation

The software requirement prioritization problem aims to determine an optimal set of software requirements that should be implemented, considering various constraints and objectives. In this section, we mathematically formulate the problem using an objective function and constraints.

#### 4.2.1. Objective Function

The primary objective is to maximize the net value derived from the selected software requirements while considering their associated costs and importance [100,101]. The objective function F is given as follows:

(23) $$F\left(x\right)=\sum_{i=1}^{n}x_{i}\times {Value}_{i}\times {Weight}_{i}-\sum_{i=1}^{n}x_{i}\times {Cost}_{i}$$

where $x_{i}$ denotes the binary decision vector, which is ‘1′ if the ith requirement is selected and ‘0′ otherwise, ${Value}_{i}$ denotes the value of the ith requirement, ${Weight}_{i}$ denotes the importance weight of the ith requirement, with the values assigned as 3 for ‘HIGH’, ‘2′ for ‘MEDIUM’, and ‘1′ for ‘LOW’ importance, ${Cost}_{i}$ denotes the cost associated with the ith requirement, and n denotes the total requirements.

#### 4.2.2. Constraints

**Budget Constraint:** The total cost of the selected requirements should not exceed the available budget B, and it is formulated as follows:

(24) $$\sum_{i=1}^{n}x_{i}\times {Cost}_{i}\leq B$$

**Prerequisite Constraint:** If a requirement has a prerequisite, it can only be selected if its prerequisite is also selected and formulated as follows:

(25) $$x_{i}\leq x_{j},\forall i,j\;such\;that\;i\;is\;a\;prerequisite\;of\;j$$

**Minimum High Importance Constraint:** At least a certain percentage $P_{H}$ of the ‘High’ importance requirements should be selected as follows:

(26) $$\sum_{i:{Imporatance}_{i}{=}^{‘}H^{’}}x_{i}\geq P_{H}\times MinHigh$$

where MinHigh is the minimum number of high-importance requirements that must be selected.

**Maximum Low Importance Constraint:** No more than a certain percentage $P_{L}$ of the ‘Low’ importance requirements should be selected as follows:

(27) $$\sum_{i:{Imporatance}_{i}{=}^{‘}L^{’}}x_{i}\leq P_{L}\times MaxLow$$

where MaxLow is the maximum number of low-importance requirements that can be selected.

The objective function aims to maximize the net benefit, which is the difference between the total value and the total cost of the selected requirements. The constraints ensure that the prerequisites of any selected requirement are also selected. The binary constraint ensures that each requirement is either selected or not.

## 5. Results and Discussions

The performance potential of the proposed algorithm is rigorously evaluated through a comprehensive set of tests and analyses. These evaluations are conducted using various methods, including:

- **23 standard benchmark functions with different dimensions:** The problems are predefined mathematical functions commonly used in optimization research to test the efficiency and accuracy of new algorithms;
- **Five engineering design problems:** These are typical problems encountered in engineering disciplines, which provide a practical context for assessing the algorithm’s applicability and effectiveness;
- **Software requirement prioritization problem:** These are intricate and multi-faceted problems sourced from real-life scenarios, offering a challenging problem for the algorithm.

All tests were conducted on a specific computer setup to ensure consistency and reliability in the evaluations. This setup was a PC running Microsoft Windows 11^®^. The hardware specification includes 16 Gigabytes of memory and an Intel(R)Core(TM)-i5 CPU with a clock speed of 2.50 GHz. For coding and executing the algorithms, MATLAB software (version 9.9 (R2020b); Massachusetts, USA) was chosen. This software is widely recognized in the research community for its versatility and robustness in handling complex mathematical computations and simulations.

When evaluating the proposed MLBRSA, it is benchmarked against several other algorithms. These include the RSA, improved RSA (IRSA) [65], reinforcement learning-based GWO (RLBGWO) [82], improved dwarf mongoose optimization algorithm (IDMOA) [102], RL-based hybrid Aquila optimizer and AOA (RLAOA) [80], adaptive gaining-sharing knowledge (AGSK) algorithm [103], and ensemble sinusoidal differential covariance matrix adaptation with Euclidean neighborhood (LSHADE-cnEpSin) algorithm [104]. The population size and the maximum number of iterations for the 23 test functions are 30 and 500, respectively, and for the real-world problems are 30 and 1000, respectively. The algorithm parameters can be found in Table A1. Each algorithm is executed 30 times, and the results are recorded for a fair comparison. The performance factors include Min, Max, Mean, Median, standard deviations (STD), run-time (RT), and Friedman’s ranking test (FRT) values.

### 5.1. Numerical Test Functions

Various statistical metrics are employed to understand each algorithm’s performance comprehensively. These metrics offer insights into the distribution, central tendency, and variability of the results. Specifically, the following metrics are presented: Minimum (Min): This represents the lowest value or score the algorithm achieves. It provides a sense of the worst-case performance; Maximum (Max): In contrast to the minimum, this metric showcases the highest value or score the algorithm achieves, indicating the best-case performance; Mean: This is the average score of the algorithm across all runs or iterations. It provides a central value that represents the typical performance of the algorithm; Standard Deviation (STD): This metric measures the amount of variation or dispersion from the mean. A low standard deviation indicates that the results are close to the mean, while a high standard deviation suggests that the results can vary widely. A specialized statistical ranking test known as the FRT is employed further to validate the performance and superiority of the MLBRSA. The FRT is a non-parametric test to detect treatment differences across multiple test attempts. The detailed findings from this test, specifically pertaining to the MLBRSA, are elaborated upon to offer a clear understanding of its standing compared to other algorithms. 

#### 5.1.1. Capacity Analysis

The benchmark functions are categorized based on their characteristics and challenges. Unimodal Benchmarks (F1–F7): These are functions with a single peak or trough. They assess an algorithm’s ability to exploit or hone in on a single optimal solution. The results for these benchmarks are tabulated in Table 1. Multi-modal Functions (F8–F13) with 30 Dimensions: Multi-modal functions have multiple peaks or troughs, making them more challenging as they test an algorithm’s exploration capability. Specifically, the ones with 30 dimensions are designed to evaluate how well an algorithm can navigate a complex search space with many variables. The results for these functions are presented in Table 2. Multi-modal Functions (F14–F23) with Fixed Dimensions: These functions also have multiple peaks or troughs but have a set number of dimensions. They are used to gauge an algorithm’s proficiency in discovering solutions in low-dimensional search spaces. The outcomes for these benchmarks are detailed in Table 3. The best results in each table are highlighted using boldface typography to make it easier for readers to identify superior performance at a glance. This visual cue ensures that standout performances are immediately recognizable.

**Figure 4:**
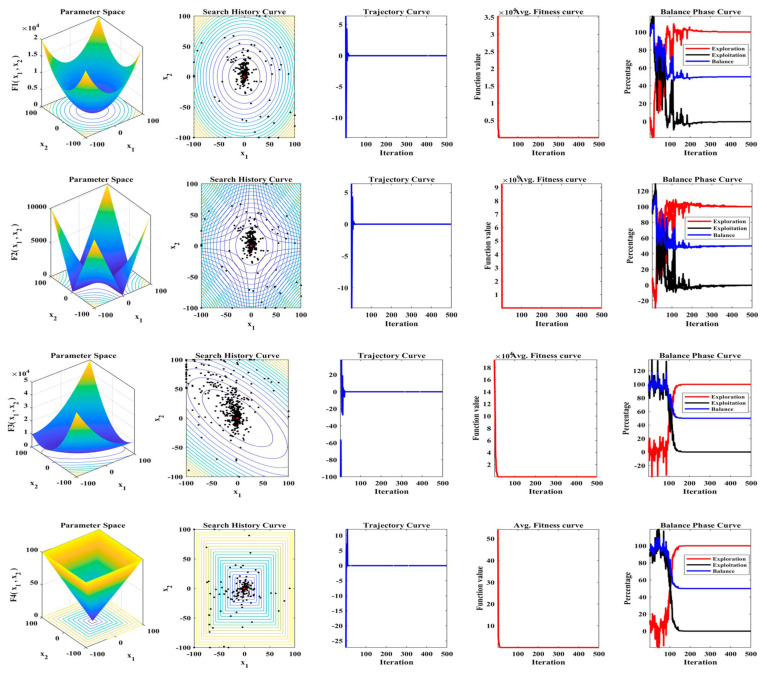

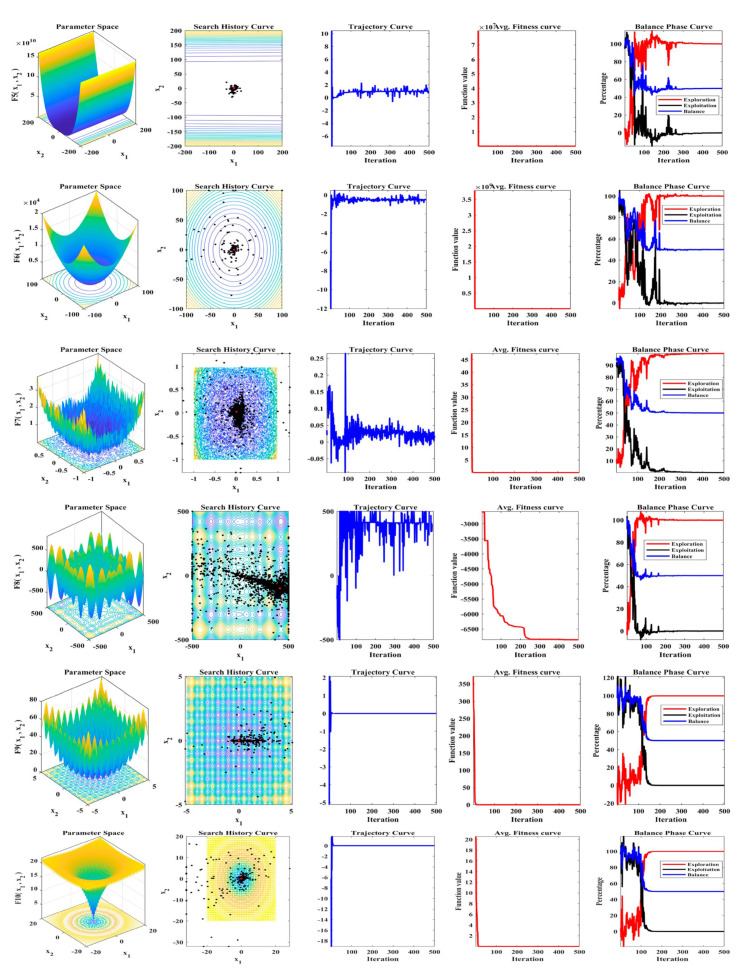

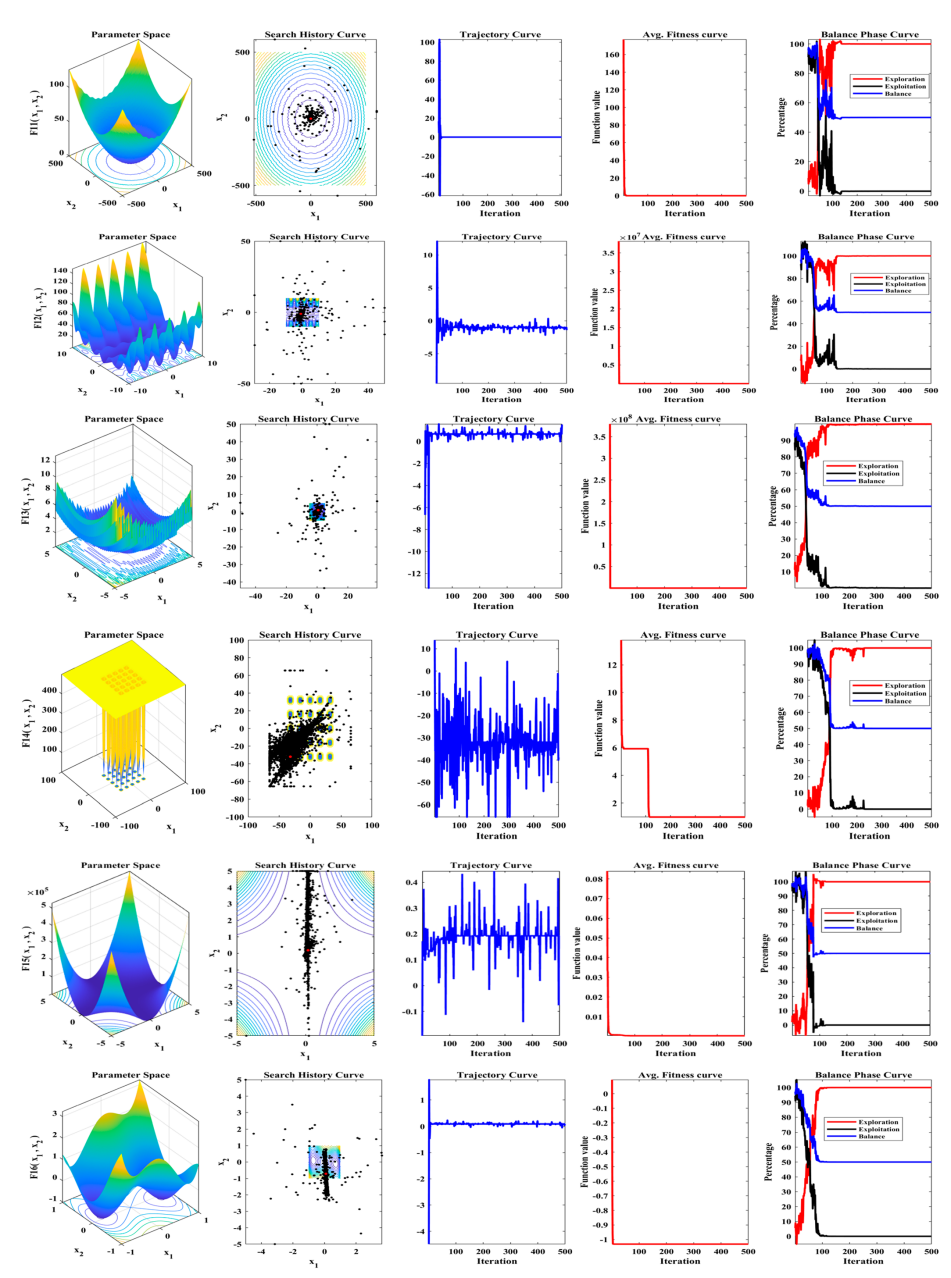

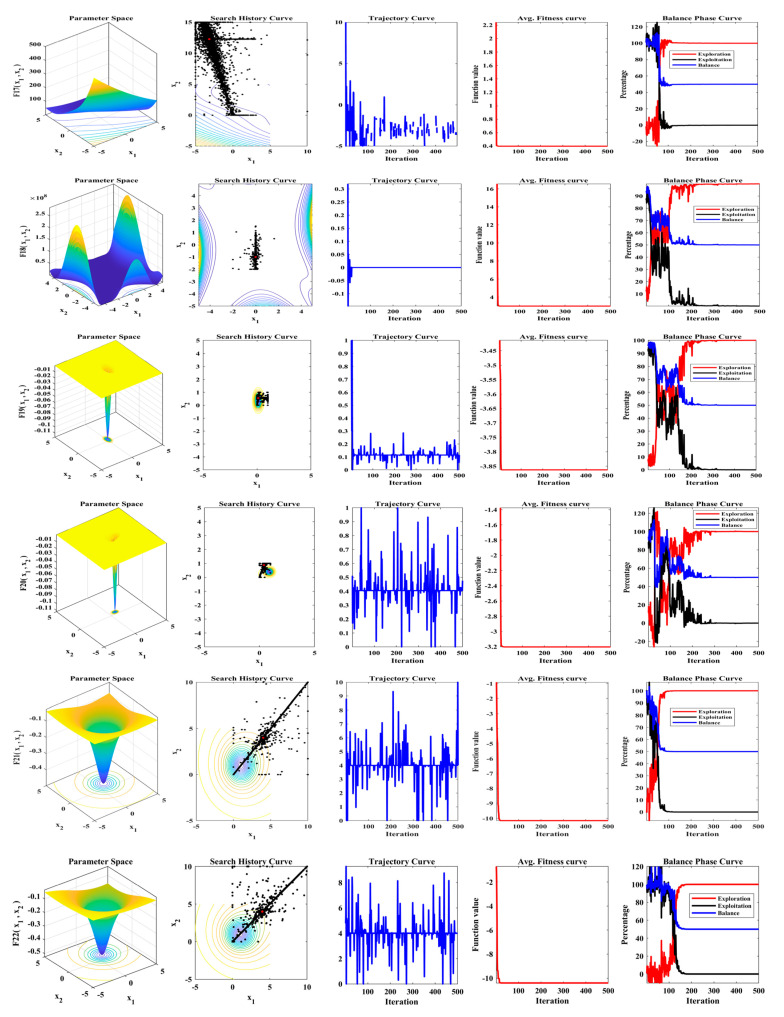

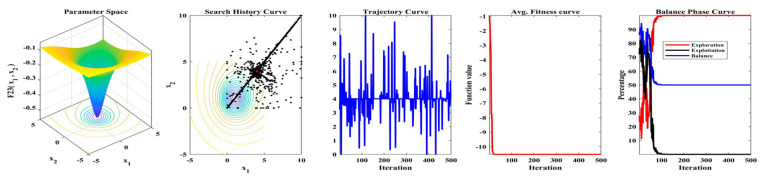
Visual representation of various metrics for numerical test functions.

Table 1, Table 2 and Table 3 provide a comprehensive overview of the performance of the proposed MLBRSA, and the results are quite impressive. Across most of the standard test functions, the MLBRSA consistently delivered optimal results. This superior performance is evident in the best results and the average and STD values, which offer insights into the central tendency and variability of the algorithm’s outcomes. Exploitation in optimization refers to an algorithm’s ability to refine its search and hone in on the best solutions in a local area. The test functions F1 through F7 serve as a measure of this capability. A closer look at Table 1 reveals that the MLBRSA emerged as the top performer in six of these seven functions. This dominance underscores the MLBRSA’s exceptional ability to exploit and find optimal solutions, outshining all other algorithms under consideration. Exploration pertains to an algorithm’s capacity to search widely across the solution space, ensuring it does not miss out on potential optimal solutions in distant regions. The test functions F8 through F13 gauge this ability. Table 2 shows that, out of these six functions, the MLBRSA surpassed other algorithms in all six, highlighting its robust exploration capabilities. The test functions F14 through F23 assess an algorithm’s proficiency in navigating low-dimensional search spaces. The MLBRSA’s prowess is also evident here, with the algorithm delivering superior results in ten functions. This demonstrates its versatility in handling both complex and simpler problem spaces. The standout performance of the MLBRSA across most test functions can be attributed to its integration of QL, competitive learning, and adaptive learning. These methodologies enhance both the exploitation and exploration abilities of the algorithm. In contrast, the RSA, which serves as a comparison, struggles due to an imbalance in its exploration and exploitation dynamics. Figure 4 provides a visual representation of various metrics across 23 test functions. Some key observations include: (i) Trajectory curve: This curve tracks the progression of the baseline parameter of the initial population over iterations. It reveals that solutions in the MLBRSA undergo significant shifts in the early phases, which taper off as the algorithm progresses. By the end, the MLBRSA stabilizes, effectively utilizing the available solution space; (ii) Mean fitness curves: These curves depict the evolution of the average fitness of the population over time, offering insights into the algorithm’s performance trajectory; (iii) Search space coverage: The MLBRSA excels in thoroughly scanning the solution space, as evident from its focus on potential solution areas in the search history; (iv) Exploratory activity: The trajectories showcase the MLBRSA’s primary exploration activities, characterized by sudden, decisive movements. This indicates the algorithm’s agility in navigating the solution space; and (v) Convergence and global best search: The MLBRSA’s ability to converge rapidly and relentless pursuit of the global best solution are also evident.

#### 5.1.2. Dimensionality Analysis

The performance of the MLBRSA is further scrutinized by examining its behavior in high-dimensional spaces. High dimensionality can pose significant challenges to optimization algorithms as the solution space grows exponentially, making it harder to find optimal solutions. The primary goal is understanding how the MLBRSA fares when confronted with large dimensions. This is crucial because the ability to handle high dimensionality is a testament to an algorithm’s robustness and versatility. Several statistical metrics are used to provide a comprehensive understanding of the performance across different algorithms. These include Min, Max, Mean, and STD. The outcomes based on these metrics for all the considered algorithms are tabulated in Table A2 (for 100 dimensions) and Table A3 (for 500 dimensions). The functions F1 through F13 are chosen for this analysis, with two distinct dimensional settings, 100 and 500. The population size for the algorithm is set at 30, and the algorithm is allowed to run for a maximum of 500 iterations. As seen in Table A2, the MLBRSA exhibits remarkable prowess. Specifically, when dealing with the functions F1–F13 set at 100 dimensions, the MLBRSA consistently outshines other algorithms. This dominance is evident across almost all the tests conducted, underscoring its ability to handle moderately high-dimensional problems easily. Moving to a much higher dimensionality, Table A3 presents the results for the 500-dimensional setting. Here, the challenge is significantly amplified due to the vastness of the solution space. Yet, the MLBRSA rises to the occasion, outperforming all other algorithms in 12 of 13 problems. This is a testament to its robust design and capabilities. The standout performance of the MLBRSA, especially in high-dimensional spaces, can be attributed to its integration of multi-learning techniques. These techniques enhance the algorithm’s ability to navigate vast solution spaces efficiently, ensuring that it does not get trapped in suboptimal solutions and continues its pursuit of the best possible outcomes. In summary, the MLBRSA’s performance in moderate (100 dimensions) and high (500 dimensions) dimensional spaces underscores its versatility and robustness. Its design, especially the incorporation of multi-learning, is pivotal in ensuring its dominance across a wide range of problems.

#### 5.1.3. Complexity Analysis 

The computation time, often referred to as the run time (RT), is a crucial metric when evaluating the efficiency of algorithms. It provides insights into how quickly an algorithm can produce results, which is especially important in real-time applications or scenarios with tight computational budgets. The primary objective is to understand the proposed MLBRSA’s computational efficiency compared to other algorithms. The RT values serve as a direct measure of this efficiency. All the RT values for the considered algorithms are systematically presented in Table A4. This table offers a side-by-side comparison, enabling readers to gauge the relative computational speeds of the algorithms quickly. A closer examination of Table A4 reveals that the mean RT for the suggested MLBRSA is 0.08 s. This is marginally higher than the basic RSA, which has an average RT of 0.18 s across all 23 test functions. This slight increase in RT for the MLBRSA might be attributed to additional features or complexities introduced in the algorithm to enhance its optimization capabilities. One reason could be the lower computational complexity inherent to the RSA. However, it is essential to note that while the RSA might be faster, its performance in terms of optimization is subpar for all the selected test functions. This highlights a trade-off between speed and optimization quality. In the grand scheme of things, the proposed MLBRSA ranks second in terms of RT. This places it behind only two other algorithms but ahead of several others. While the MLBRSA might not be the fastest in terms of computation time, it is essential to consider the balance between speed and optimization performance. An algorithm might be swift but not provide the best optimization results, making the slight increase in RT for better performance a worthy trade-off in many scenarios. In summary, while the proposed MLBRSA might take slightly longer to compute than others, its superior optimization capabilities make it a valuable choice. The detailed analysis of RT values underscores the importance of considering speed and quality when evaluating optimization algorithms.

#### 5.1.4. Statistical Test Analysis

The evaluation of algorithms often necessitates a rigorous statistical approach to ensure that the observed results are valid and reliable. One of the primary tools in this regard is the statistical rank test. This test is essential to rank and compare algorithms based on their observed performance metrics. By doing so, researchers can determine which algorithm is superior in specific contexts or under certain conditions. The FRT is a prominent choice among the various statistical rank tests available. It is renowned and widely adopted in research circles for its efficacy in ranking algorithms. The FRT is a non-parametric test, which means it does not assume a specific distribution for the underlying data. This makes it versatile and applicable to a wide range of datasets. It is an alternative to the one-way ANOVA, which compares means across different groups. The FRT is particularly suitable when the parameter under evaluation is continuous. It is designed to detect differences or variations across multiple groups or sets. A critical aspect of any statistical test is the significance level, which is set at 0.05 in this context. This means that there is a 5% risk of concluding that a difference exists when, in reality, there is not. If the *p*-value (a measure of the evidence against a null hypothesis) obtained from the test is less than or equal to this significance level, the null hypothesis is rejected. In simpler terms, if the *p*-value is 0.05 or less, it suggests that not all group median values are the same. This study employs the FRT as the primary tool to rank the algorithms. This choice underscores the trust the research community places in the FRT for such evaluations. Table A5 provides a comprehensive overview of the FRT values for all the algorithms across the 23 test functions under consideration. This table lists individual FRT values and presents the average FRT values, which are pivotal in the ranking process. In summary, the FRT is a robust and reliable tool for ranking algorithms in this research. By comparing the FRT values and using a stringent significance level, the study ensures that the rankings are valid and scientifically sound. The detailed presentation of these values in Table A5 further aids in transparency and clarity, allowing readers to understand the relative performance of each algorithm.

#### 5.1.5. Convergence Analysis

**Figure 5:**
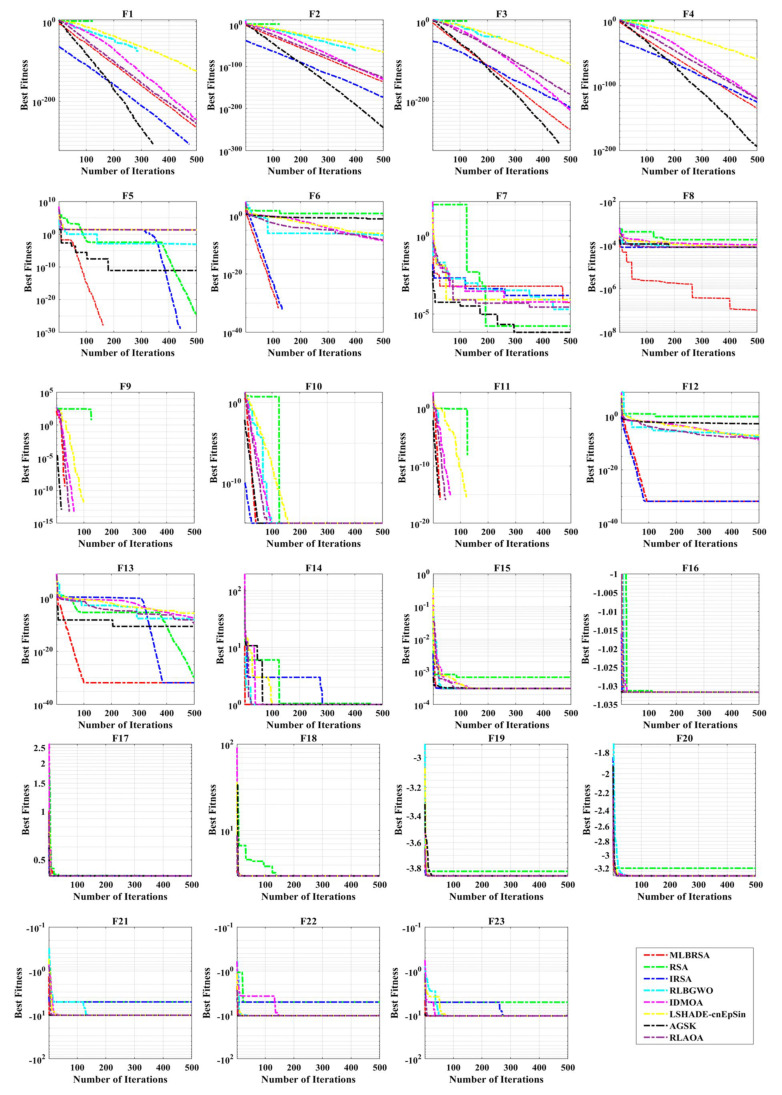
Convergence curves of all selected algorithms.

**Figure 6:**
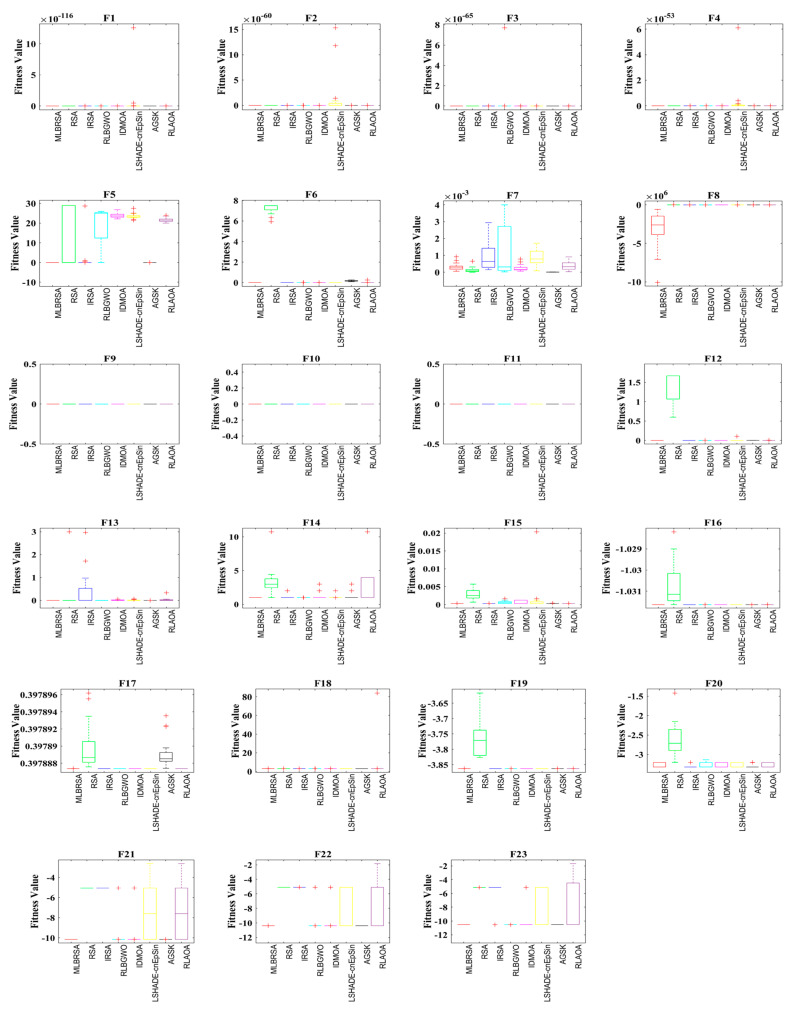
Box plots of the selected algorithms.

The performance of the MLBRSA, particularly its convergence activities, has been meticulously studied. Convergence in optimization refers to the algorithm’s ability to approach and find the optimal solution over iterations. The primary goal was to understand and evaluate the highest score metric of the MLBRSA, specifically its ability to converge to the optimal value. This optimal value is a benchmark to gauge how close the algorithm gets to the best possible solution. The speed at which the MLBRSA converges to the optimal solution was analyzed for every benchmark function used in the study. This speed is a testament to the algorithm’s efficiency and ability to find solutions quickly. In order to provide a comprehensive perspective on the MLBRSA’s performance, it was benchmarked against several other algorithms. The performance metrics were obtained over 30 runs to ensure reliability and consistency in the results. Figure 5 provides a visual representation of the convergence rates of the various algorithms. The MLBRSA, in most scenarios, showcases a commendable convergence rate, often outpacing other methods. This is indicative of its robust design and optimization capabilities. The results highlight the synergistic effect of integrating a multi-learning strategy with the RSA. This integration has led to a marked enhancement in the convergence efficiency of the optimization algorithm. Not only does the MLBRSA converge faster than other algorithms, but it also reaches the optimal value in fewer iterations. This rapid convergence rate sets it apart from other techniques, emphasizing its efficiency. Box plots are graphical tools that visually represent data distribution through quartiles, depicting five key statistical metrics: the minimum, first quartile (25th percentile), median (50th percentile), third quartile (75th percentile), and maximum. These plots provide insights into the data’s spread, symmetry, and central tendency. For all 23 benchmark functions, box plots were generated for each selected algorithm. These are visually presented in Figure 6, offering a detailed view of the proposed algorithm’s data distribution characteristics. It showcases the symmetry, spread, and centrality of the MLBRSA’s performance metrics. A closer look at Figure 6 reveals that the statistical attributes of the MLBRSA surpass those of all other algorithms under consideration. 

Finally, the MLBRSA’s convergence capabilities have been thoroughly analyzed and benchmarked against other algorithms. Its rapid convergence rate and ability to achieve optimal values in fewer iterations underscore its superiority. The visual representations provided by the box plots further emphasize its standout performance across various benchmark functions.

### 5.2. Engineering Design Optimization Problems

In this sub-section, we delve into evaluating the performance of the newly introduced MLBRSA. This evaluation is done by applying it to five specific engineering design challenges. These challenges include (i) welded beam design, (ii) pressure vessel design, (iii) tension/compression spring design, (iv) three-bar truss design, and (v) tubular column design problems. 

Each design problem has its constraints, making them particularly challenging. The primary reason for choosing these specific problems is to rigorously test the capability of the MLBRSA in effectively managing and solving constrained optimization challenges. To ensure a comprehensive assessment, each algorithm, including the proposed MLBRSA, is run individually a total of 30 times. For every run, a consistent population size of 30 is maintained. The maximum iteration count for all these algorithms is also capped at 1000. One of the significant challenges in optimization problems is managing constraints. This study has employed the static penalty constraint handling mechanism to address this [105]. This mechanism aids in ensuring that the constraints are adhered to during the optimization process. It is essential to note that the objective functions chosen for all the design mentioned above problems are geared towards minimization. In other words, these optimization problems aim to find the smallest possible value that satisfies all the given constraints. 

#### 5.2.1. Welded Beam Design Problem

**Figure 7:**
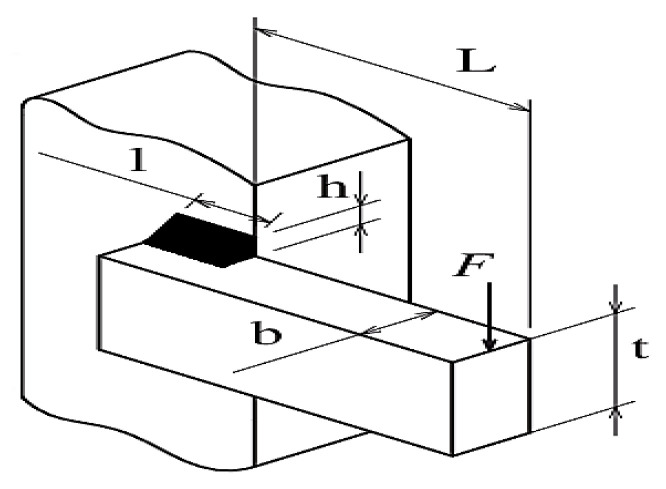
Structure of the welded beam design.

The main objective of the welded beam design problem is to identify the optimal cost while considering the constraints. The problem considers four design variables $x=[x_{1},x_{2},x_{3},x_{4}]$, i.e., [h,l,t,b], in which l defines the length, b defines bar thickness, t defines the weld thickness, and h defines the height. The welded design problem has five equality constraints such as beam blending stress (θ), shear stress (τ), bar buckling load $\left(P_{c}\right)$, beam end deflection (δ), and side constraints. The upper bounds and lower bounds of all design variables are $0.1⩽x_{1}⩽2$, $0.1⩽x_{2}⩽10$, $0.1⩽x_{3}⩽10$, and $0.1⩽x_{4}⩽2$. In addition, other design variables are selected as $\sigma_{max}=30,000\;\mathrm{psi}$, $\tau_{max}=13,600\;\mathrm{psi},$ $G=12\times 10^{6}\;\mathrm{psi},$ $E=30\times 1^{6}\;\mathrm{psi},$ $\delta_{max}=0.25\;\mathrm{in}.,$ L=14 in., and P=6000 lb. The welded beam design is illustrated in Figure 7. The fitness function and the constraints of the welded beam design problem are as follows [106]:

(28) $$f_{1}\left(x\right)=1.10471{\;x}_{1}^{2}x_{2}+0.04811\;x_{3}x_{4}\left(14+x_{2}\right)$$

subjected to:

(29) $$\left.\begin{array}{c} g_{1}\left(\overset{\to }{x}\right)=\tau \left(\overset{\to }{x}\right)-\tau_{max}⩽0\\ g_{2}\left(\overset{\to }{x}\right)=\sigma \left(\overset{\to }{x}\right)-\sigma_{max}⩽0\\ g_{3}\left(\overset{\to }{x}\right)=\delta \left(\overset{\to }{x}\right)-\delta_{max}⩽0\\ g_{4}\left(\overset{\to }{x}\right)=x_{1}-x_{4}⩽0\\ g_{5}\left(\overset{\to }{x}\right)=P-P_{c}\left(\overset{\to }{x}\right)⩽0\\ g_{6}\left(\overset{\to }{x}\right)=0.125-x_{1}⩽0\\ g_{7}\left(\overset{\to }{x}\right)=1.10471x_{1}^{2}+0.04811x_{3}x_{4}\left(14.0+x_{2}\right)-5.0⩽0 \end{array}\right\}$$

The results obtained by the MLBRSA and other algorithms, such as the IRSA, RSA, RLBGWO, IDMOA, LSHADE-cnEpSin, AGSK, and RLAOA, are listed in Table 4. Table 4 shows that the MLBRSA outperformed all of the other approaches and cost the least. Table 4 additionally includes statistical information such as the Min, Mean, STD, and RT. As a result, it is decided that the suggested MLBRSA is more reliable for the welded beam design optimization problem. The convergence curves and boxplot analysis of all algorithms are shown in Figure 12b and Figure 13b. Furthermore, all FRT values derived by all algorithms are presented. The proposed MLBRSA comes out on top when it comes to solving the welded beam design challenge.

#### 5.2.2. Pressure Vessel Design Problem

**Figure 8:**
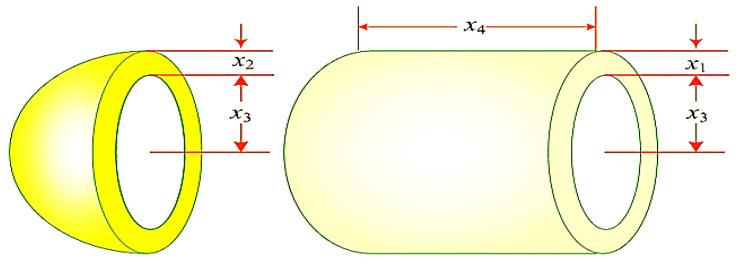
Structure of pressure vessel design.

Figure 8 depicts the schematic of the pressure vessel design optimization problem. The pressure vessel features capped ends and hemispherical heads. Minimization of construction costs is the primary objective of this problem. It considers four control vectors $x=\left[x_{1},x_{2},x_{3},x_{4}\right]=[T_{s},T_{h},R,L]$, where $T_{s}$ denotes the shell thickness, $T_{h}$ denotes the head thickness, R denotes the inner radius, and L denotes the cylindrical section length. This problem also has four equality constraints, as listed in Equation (31). The bounds of variables are $0\leq T_{s},{\;T}_{h}\leq 99$ and 10≤R,  L≤200. Equation (30) denotes the primary objective of the pressure vessel design problem [106]:

(30) $$f_{2}\left(x\right)=0.6224\;x_{1}x_{3}x_{4}+1.7781\;x_{2}x_{3}^{2}+3.1661\;x_{1}^{2}x_{4}+19.84\;x_{1}^{2}x_{3}$$

subjected to:

(31) $$\left.\begin{array}{c} g_{1}\left(x\right)=-x_{1}+0.0193x\\ g_{2}\left(x\right)=-x_{2}+0.00954x_{3}\leq 0\\ g_{3}\left(x\right)=-\pi x_{3}^{2}x_{4}-\left(4/3\right)\pi x_{3}^{3}+1,296,000\leq 0\\ g_{4}\left(x\right)=x_{4}-240\leq 0 \end{array}\right\}$$

The results obtained by the MLBRSA and other algorithms, such as the IRSA, RSA, RLBGWO, IDMOA, LSHADE-cnEpSin, AGSK, and RLAOA, are listed in Table 5. Table 5 shows that the MLBRSA outperformed all of the other approaches, and the obtained cost is minimal compared to other algorithms. Table 5 includes statistics such as the Min, Mean, STD, and RT. As a result, it is decided that the suggested MLBRSA is a reliable tool for the pressure vessel design optimization problem. The convergence curves and boxplot analysis of all algorithms are shown in Figures 12b and 13b. Furthermore, all FRT values derived by all algorithms are presented. The proposed MLBRSA comes out on top when it comes to solving the welded beam design challenge.

#### 5.2.3. Tension/Compression Spring Design Problem

**Figure 9:**
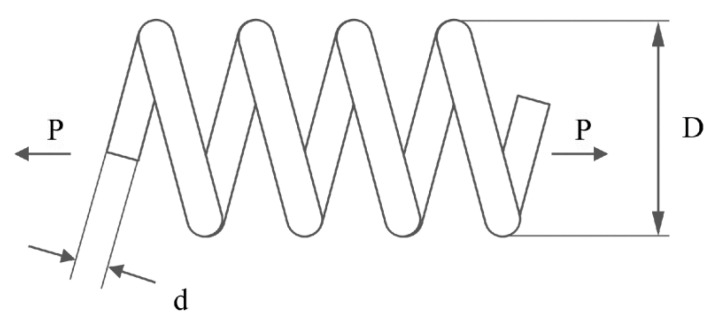
Structure of the tension/compression spring design.

Another classic mechanical engineering design that has been considered is the tension/compression spring design. The main objective of the spring design problem is to reduce the tension spring weight of the framework, and the structure is depicted in Figure 9. It considers three control vectors $x=\left[x_{1},x_{2},x_{3}\right]=[d,D,N]$, where D denotes the mean coil dia, d denotes the wire dia, and N denotes active coils. This problem also has four equality constraints, as listed in Equation (33). The bounds of variables are 0.05≤d≤2, 0.25≤D≤1.3, and 2≤N≤15. Equation (32) denotes the primary objective of the tension/compression spring design problem [106]:

(32) $$f_{3}\left(\vec{x}\right)=\left(x_{3}+2\right)x_{2}x_{1}^{2}$$

subjected to:

(33) $$\left.\begin{array}{c} g_{1}\left(\vec{x}\right)=1-\frac{x_{2}^{3}x_{3}}{71785x_{1}^{4}}\leq 0\\ g_{2}\left(\vec{x}\right)=\frac{4x_{2}^{2}-x_{1}x_{2}}{12566\left(x_{2}x_{1}^{3}-x_{1}^{4}\right)}+\frac{1}{5108x_{1}^{2}}-1\leq 0\\ g_{3}\left(\vec{x}\right)=1-\frac{140.45x_{1}}{x_{2}^{2}x_{3}}\leq 0\\ g_{4}\left(\vec{x}\right)=\frac{x_{1}+x_{2}}{1.5}-1\leq 0 \end{array}\right\}$$

The results obtained by the MLBRSA and other algorithms, such as the IRSA, RSA, RLBGWO, IDMOA, LSHADE-cnEpSin, AGSK, and RLAOA, are listed in Table 6. Table 6 shows that the MLBRSA outperformed all of the other approaches, and the obtained weight is minimal compared to other algorithms. Table 6 includes statistics such as the Min, Mean, STD, and RT. As a result, it is decided that the suggested MLBRSA is a reliable tool for the tension/compression design optimization problem. The convergence curves and boxplot analysis of all algorithms are shown in Figures 12b and 13b. Furthermore, all FRT values derived by all algorithms are presented. The proposed MLBRSA comes out on top when it comes to solving the tension/compression spring design challenge.

#### 5.2.4. Three-Bar Truss Design Problem

**Figure 10:**
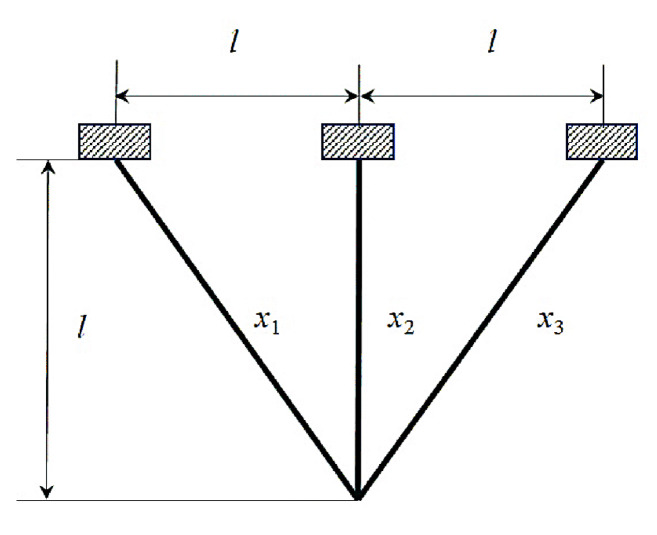
Structure of the three-bar truss design.

The primary objective of the three-bar truss design is to reduce the weight of the bar constructions. The problem has three equality constraints, including each bar’s stress, buckling, and deflection. The problem has three control vectors: $x=\left[x_{1},x_{2},x_{3}=x_{1}\right]=[A_{1},A_{2}]$. The bounds of variables are $0\leq x_{1},x_{2},x_{3}\leq 1,$ and the values of a few other parameters are l=100 cm, P=2 kN/cm^2^, and σ=2 kN/cm^2^. The primary objective is presented in Equation (34), and the equality constraints are listed in Equation (35). The structure of the three-bar truss is shown in Figure 10 [106].

(34) $$f_{4}\left(\vec{x}\right)=\left(2\sqrt{2}x_{1}+x_{2}\right)\;*l\;$$

subject to:

(35) $$\left.\begin{array}{c} g_{1}\left(\vec{x}\right)=\frac{2\sqrt{2}x_{1}+x_{2}}{\sqrt{2}{x_{1}}^{2}+2{x_{1}x}_{2}},\;P-\sigma \leq 0\\ g_{2}\left(\vec{x}\right)=\frac{x_{2}}{\sqrt{2}{x_{1}}^{2}+2{x_{1}x}_{2}},\;P-\sigma \leq 0\\ g_{3}\left(\vec{x}\right)=\frac{1}{\sqrt{2}x_{2}+x_{1}},\;P-\sigma \leq 0 \end{array}\right\}$$

The results obtained by the MLBRSA and other algorithms, such as the IRSA, RSA, RLBGWO, IDMOA, LSHADE-cnEpSin, AGSK, and RLAOA, are listed in Table 7. Table 7 shows that the MLBRSA outperformed all of the other approaches, and the obtained weight is minimal compared to other algorithms. Table 7 includes statistics such as the Min, Mean, STD, and RT. As a result, it is decided that the suggested MLBRSA is a reliable tool for the three-bar truss design optimization problem. The convergence curves and boxplot analysis of all algorithms are shown in Figures 12b and 13b. Furthermore, FRT values derived by all algorithms are presented. The proposed MLBRSA comes out on top when it comes to solving the welded beam design challenge.

#### 5.2.5. Tubular Column Design Problem

**Figure 11:**
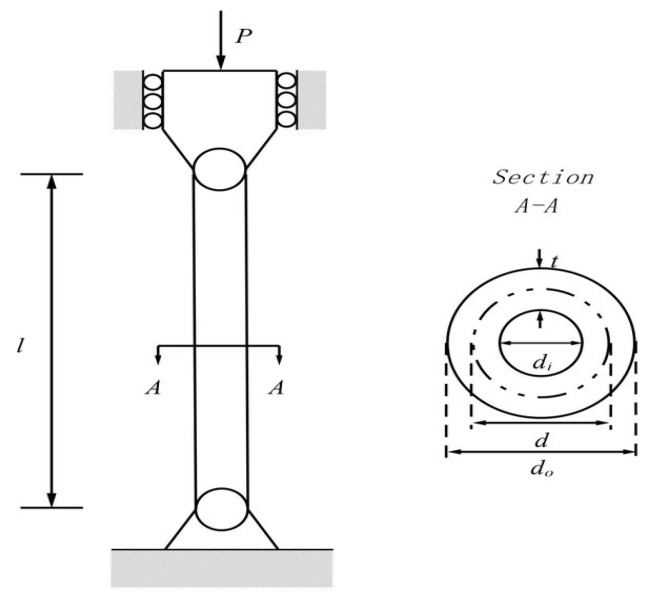
Structure of the tubular column design.

To handle a compressive load P of 2500 kgf for the least cost, a uniform column of the tubular section should be built with hinge joints at both ends. The structure of the tubular column design is depicted in Figure 11. The material used to make the column has a yield strength ($\sigma_{y}$) of 500 $\mathrm{kgf}/{\mathrm{cm}}^{3}$, an elastic modulus (E) of $0.85\;\times \;106\;\mathrm{kgf}/{\mathrm{cm}}^{2}$, and a weight density (ρ) of $0.0025\;\mathrm{kgf}/{\mathrm{cm}}^{3}$. The column measures 250 cm in length (L). The column’s stress should be less than the yield stress (constraint $g_{1}$) and buckling stress, respectively (constraint $g_{2}$). The column’s average diameter is limited to being between 2 and 14 cm, and columns thicker than 0.2 to 0.8 cm are not readily accessible on the market. The cost of the column is expressed as 5 W+ 2d, where W is the weight in kg of force and d is the average diameter of the column in centimetres. The objective function is presented in Equation (36), and six equality constraints are listed in Equation (37). It considers two control vectors $x=\left[x_{1},x_{2}\right]=[d,t]$ [106]:

(36) $$f_{5}\left(\vec{x}\right)=9.8\times d\times t+2\times d$$

subject to:

(37) $$\left.\begin{array}{c} \begin{array}{c} g_{1}\left(\vec{x}\right)=\frac{P}{\pi dt}\leq \sigma_{y}\\ g_{2}\left(\vec{x}\right)=\frac{P}{\pi dt}-\frac{\pi^{2}E\left(d^{2}+t^{2}\right)}{8L^{2}}\leq 0\\ g_{3}\left(\vec{x}\right)=-d+2\leq 0 \end{array}\\ g_{4}\left(\vec{x}\right)=d-14\leq 0\\ g_{5}\left(\vec{x}\right)=-t+0.2\leq 0\\ g_{6}\left(\vec{x}\right)=t-0.8\leq 0 \end{array}\right\}$$

**Figure 12:**
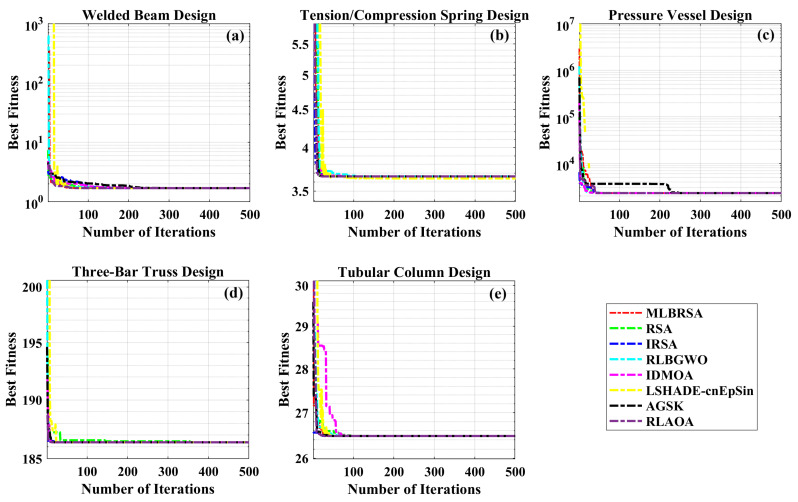
Convergence curves of all algorithms: (**a**) Welded beam design, (**b**) Pressure vessel design, (**c**) Tension/compression spring design, (**d**) Three-bar truss design, (**e**) Tubular column design.

**Figure 13:**
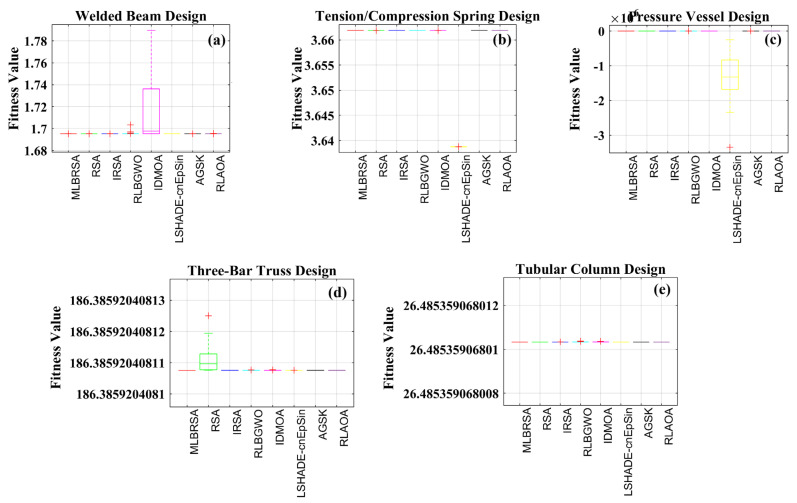
Boxplots of all algorithms; (**a**) Welded beam design, (**b**) Pressure vessel design, (**c**) Tension/compression spring design, (**d**) Three-bar truss design, and (**e**) Tubular column design.

The results obtained by the MLBRSA and other algorithms, such as the IRSA, RSA, RLBGWO, IDMOA, LSHADE-cnEpSin, AGSK, and RLAOA, are listed in Table 8. Table 8 shows that the MLBRSA outperformed all of the other approaches, and the obtained cost is minimal compared to other algorithms. Table 8 includes statistics such as the Min, Mean, STD, and RT. As a result, it is decided that the suggested MLBRSA is a reliable tool for the tubular column design optimization problem. The convergence curves and boxplot analysis of all algorithms are shown in Figure 12b and Figure 13b. Furthermore, FRT values derived by all algorithms are presented. The proposed MLBRSA comes out on top when it comes to solving the tubular column design challenge.

### 5.3. Results Obtained for SRP Problem

The software requirement prioritization problem is a multifaceted challenge that demands a balance between various factors such as cost, value, and importance. In this section, we dissect the results obtained from the RSA and its enhanced version, i.e., the MLBRSA, to understand their efficacy in addressing this challenge. This study ignored the comparison among other peers, such as the RLBGWO, IDMOA, LSHADE-cnEpSin, AGSK, and RLAOA. The comparison is unfair because none of the selected algorithms are utilized for this objective. Therefore, this study considered the original RSA and the proposed MLBRSA to prove the superiority of the MLBRSA over the RSA, but not others. The RSA and MLBRSA are applied to the SRP problem directly. Each algorithm is executed 30 times individually for a fair comparison. The population size and the maximum number of iterations are selected as 30 and 100, respectively. All other parameters of the RSA and MLBRSA are selected as per the previous discussions. The data required for the SRP is available in [105].

**Figure 14:**
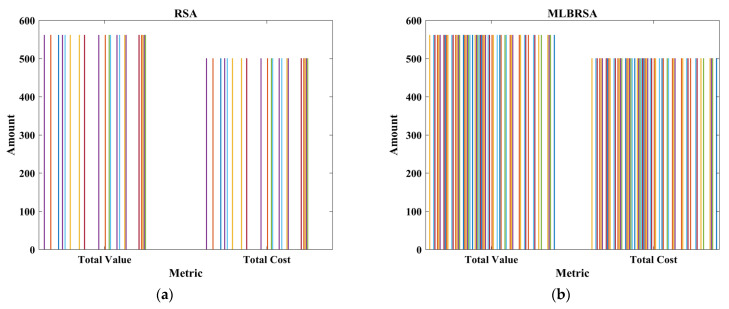
Balance between the value and the cost; (**a**) RSA; (**b**) MLBRSA.

**Figure 15:**
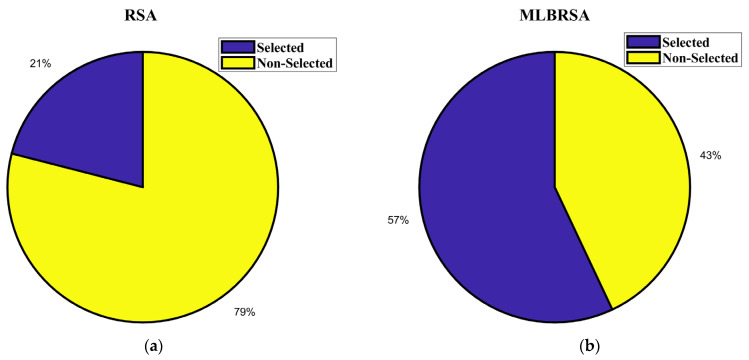
Selected and non-selected requirements; (**a**) RSA; (**b**) MLBRSA.

Firstly, the bar graph between the total value and cost is shown in Figure 14. The bar graph offers a clear visual representation of the balance RSA strikes between value and cost. While the RSA does manage to select requirements that offer value, it occasionally overshoots the budget, suggesting potential inefficiencies or a lack of stringent adherence to budget constraints. The performance of the MLBRSA is notably superior. The algorithm consistently zeroes in on requirements that maximize value while ensuring costs are kept within the stipulated budget. This demonstrates the efficacy of the proposed strategy. Figure 15 shows the pie chart between the proportion of selected and non-selected requirements obtained by both the RSA and MLBRSA. The pie chart reveals the RSA’s inclination to select a substantial portion of the available requirements. This might indicate a broader, less discriminating selection approach, which could include less critical requirements at the expense of more pivotal ones. The proposed MLBRSA showcases a more discerning selection process. The algorithm’s focus on high-value and high-importance requirements ensures that the selections are more attuned to project priorities.

**Figure 16:**
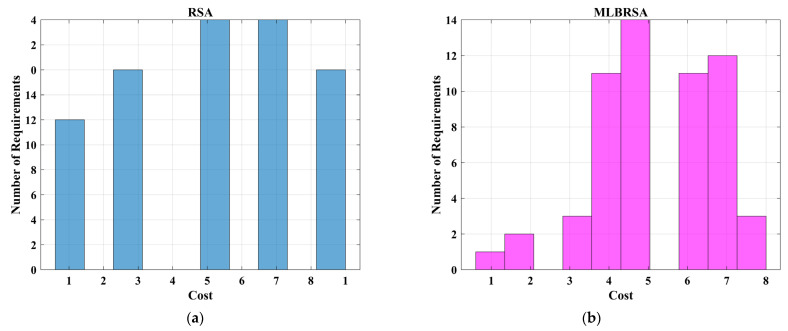
Cost distributions for the selected requirements; (**a**) RSA; (**b**) MLBRSA.

**Figure 17:**
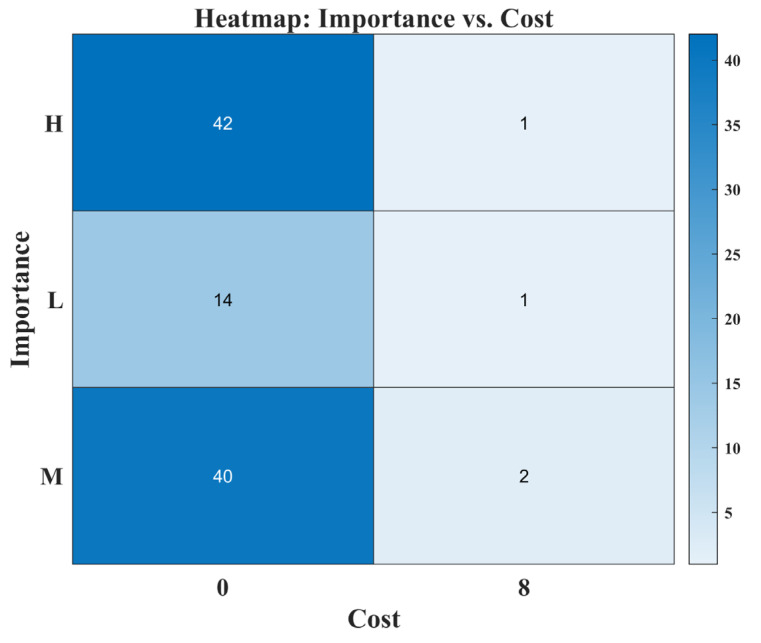
Heatmap generated between cost and importance.

Figure 16 shows the distribution of the costs for the selected requirements obtained by the RSA and MLBRSA. The histogram shows the diversity in the costs of the requirements chosen by the RSA. While diversity is commendable, the spread suggests that the algorithm might not always prioritize the most value-driven requirements. The proposed MLBRSA leans towards higher-value requirements, even if they are associated with a slightly elevated cost. This suggests a more value-centric selection approach, which is crucial for projects with tight budgets. Figure 17 shows the heatmap of the importance and cost. The original RSA’s heatmap indicates a somewhat scattered approach. The algorithm sometimes leans towards medium and low-importance requirements, even with a higher price tag. This could lead to suboptimal selections when budget constraints are tight. The proposed MLBRSA’s heatmap is evidence of its refined selection process. The pronounced selection of high-importance requirements, even those with steeper costs, aligns perfectly with the proposed strategies’ focus on importance.

**Figure 18:**
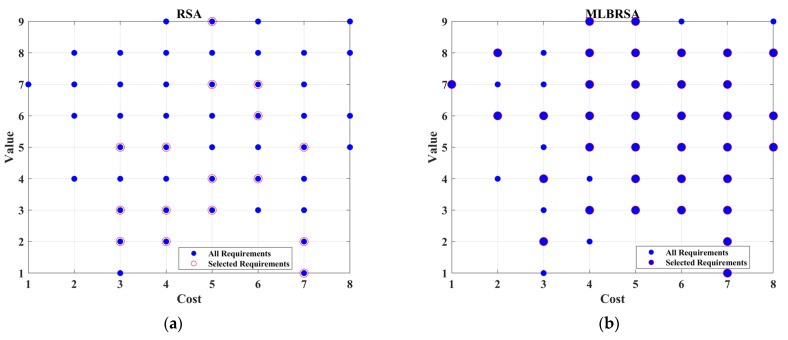
Value and cost of the selected requirements; (**a**) RSA; (**b**) MLBRSA.

**Figure 19:**
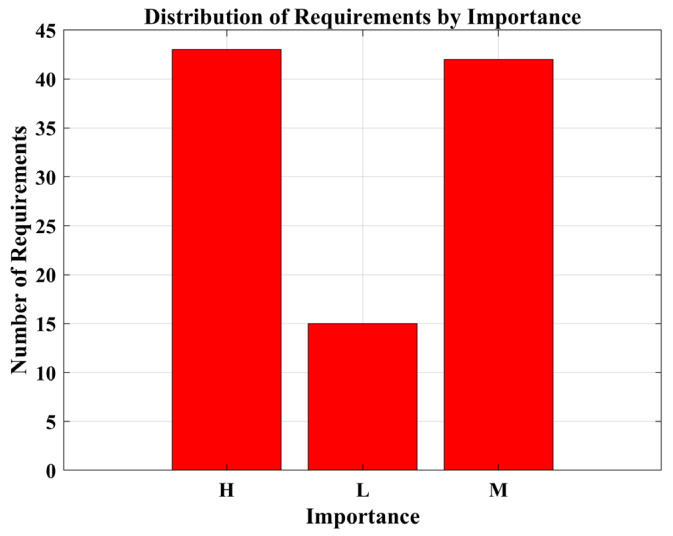
Requirement distribution by importance.

**Figure 20:**
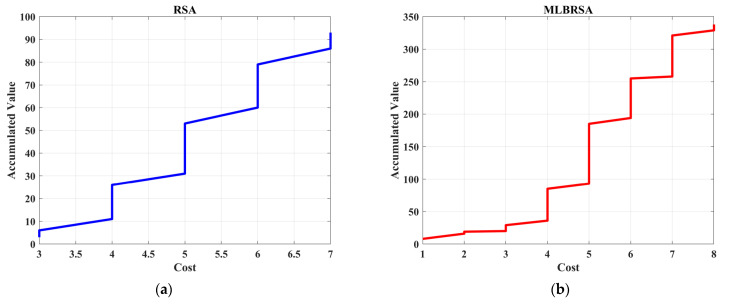
Cost and accumulated values; (**a**) RSA; (**b**) MLBRSA.

Figure 18 shows the scatter plot between the value and the cost. While the RSA’s selections are dispersed, the MLBRSA’s choices cluster around high-value requirements. This clustering indicates the MLBRSA’s capability to identify and prioritize high-value requirements consistently. Figure 19 shows the distribution by importance, and Figure 18 shows the ability of the proposed MLBRSA to prioritize ‘High’ importance requirements, further emphasizing its alignment with project priorities. Figure 20 shows the line graph of cost and the accumulated value. The proposed algorithm obtained a steeper curve, and it is indicative of its efficiency. The algorithm accumulates value at a faster rate relative to cost, showcasing its prowess in maximizing value while being cost-effective.

**Figure 21:**
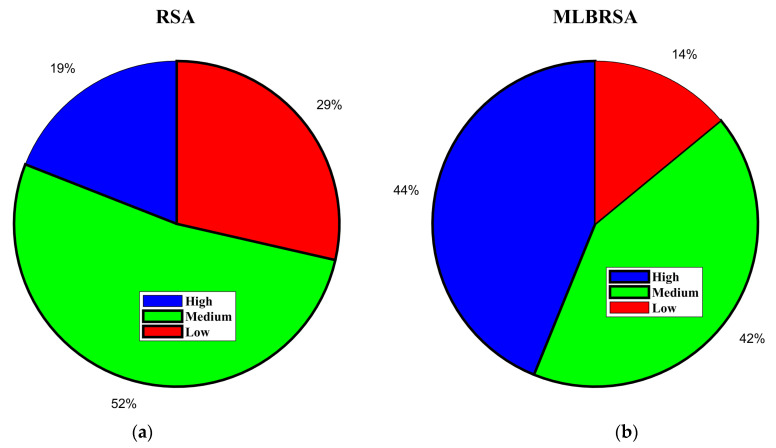
Importance distribution for the selected requirements; (**a**) RSA; (**b**) MLBRSA.

Figure 21 shows the important distribution of selected requirements. The pie chart for RSA reveals a somewhat even distribution across the importance categories. While this might suggest a balanced approach, it also indicates that the RSA might not emphasize high-importance requirements, which are crucial for the project’s success. In contrast, the proposed MLBRSA significantly emphasizes high-importance requirements. This is a testament to the algorithm’s refined selection process, which prioritizes requirements deemed critical for the project. 

**Figure 22:**
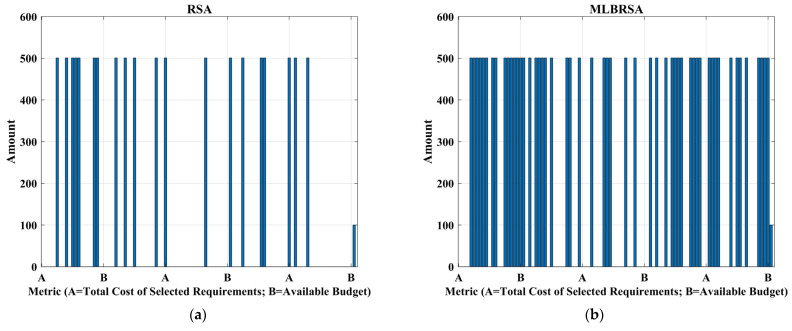
Budget utilization; (**a**) RSA; (**b**) MLBRSA.

**Figure 23:**
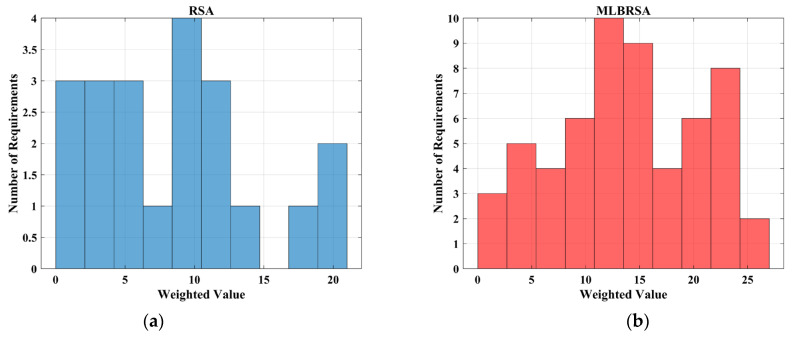
Weighted values of selected requirements; (**a**) RSA; (**b**) MLBRSA.

Figure 22 shows the budget utilization. It demonstrates the prowess of the proposed MLBRSA in budget management. Not only does it ensure that the selected requirements offer maximum value, but it also ensures that the total cost remains within the stipulated budget. This is crucial for projects where budget adherence is non-negotiable. Figure 23 shows the histogram for weighted values of selected requirements. This histogram provides a deeper insight into the value-centric approach of the MLBRSA. The pronounced peaks in the higher weighted value regions indicate that the MLBRSA consistently selects requirements that offer the best value for money. This aligns with the objective function’s focus on maximizing weighted value.

In summary, the comparative analysis is discussed as follows: (i) Value Maximization: Across all visual representations, the MLBRSA consistently outshines RSA in terms of maximizing value. This is particularly evident in Figure 14, Figure 18 and Figure 20; (ii) Budget Adherence: The MLBRSA’s stringent adherence to budget constraints, as seen in Figure 14, sets it apart from the RSA. Figure 20 underscores the MLBRSA’s unmatched ability to maximize value while ensuring strict adherence to the budget; (iii) Importance Consideration: Figure 17 and Figure 18 highlight the superior capability of the MLBRSA to prioritize and select high-importance requirements. Figure 19 showcases the MLBRSA’s superior capability to prioritize and select high-importance requirements. While the RSA offers a balanced approach, the MLBRSA’s focus on high-importance requirements ensures that the project’s critical needs are addressed; (iv) Efficiency in Value Accumulation: Figure 19 showcases the MLBRSA’s unmatched efficiency in rapidly accumulating value relative to cost; and (v) Value-Centric Approach: Figure 23 provides compelling evidence of the MLBRSA’s value-driven selection process. The pronounced peaks in the higher weighted value regions indicate the MLBRSA’s ability to identify and prioritize high-value requirements consistently.

The visual representations prove the superiority of the proposed MLBRSA in addressing the software requirement prioritization problem. While the RSA offers a broad-based approach, the MLBRSA’s refined objective function and additional constraints ensure a more targeted, value-driven, and budget-conscious selection process. The RSA’s broader selection might suit projects with flexible budgets and less stringent requirement priorities. However, for projects where every dollar counts and priorities are non-negotiable, the MLBRSA’s discerning and value-centric approach is invaluable. The results offer a clear visual representation of how both algorithms prioritize importance. While the RSA’s balanced approach might seem commendable, the proposed MLBRSA’s emphasis on high-importance requirements aligns better with the project’s critical needs. Budget management is another area where the proposed MLBRSA shines.

Projects often grapple with budget constraints, making it imperative for the selection process to offer maximum value without overshooting the budget. In addition, the provided results also offer a deep dive into the value-centric approach of the algorithms. The RSA’s selections, while valuable, often do not offer the best value for money. The proposed MLBRSA, with its pronounced peaks in the higher weighted value regions, consistently zeroes in on requirements that offer the best bang for the buck. In conclusion, the visual representations provide compelling evidence of the proposed MLBRSA’s refined, value-driven, and budget-conscious selection process. For projects where importance prioritization, budget adherence, and value maximization are paramount, the proposed MLBRSA emerges as the clear winner.

## 6. Conclusions

The introduction of the Multi-Learning-Based Reptile Search Algorithm (MLBRSA) marks a pivotal moment in the landscape of computational problem-solving. By seamlessly intertwining the principles of QL, competitive learning, and adaptive learning, the MLBRSA emerges as an encouragement of modernization, setting a new benchmark for algorithmic efficiency and versatility. Its inherent design, which capitalizes on reinforcement, competition, and adaptability, equips it with a unique prowess to delve deep into complex problem terrains and extract optimal solutions. By amalgamating the principles of QL, competitive learning, and adaptive learning, the MLBRSA not only addresses the inherent challenges posed by complex engineering problems but also excels in the domain of software requirement prioritization. Its unique ability to combine reinforcement, competition, and adaptability ensures it can navigate intricate problem spaces, continually refining its solutions. The empirical validations, as evidenced by its applications to numerical benchmarks and real-world engineering problems, not only validate its theoretical soundness but also highlight its practical relevance. In the software development sphere, where the prioritization of requirements is often a daunting task fraught with uncertainties, the MLBRSA performed well. In the context of software development, the algorithm’s proficiency at ranking requirements ensures that pivotal software functionalities receive the attention they warrant, thereby optimizing the development process. It offers a systematic, experience-driven approach to ensure that pivotal software functionalities are not just recognized but also prioritized, optimizing the overall development trajectory.

Looking ahead, the potential applications of the MLBRSA are vast. Given its demonstrated proficiency, it can be extended to other domains, such as artificial intelligence, robotics, and bioinformatics. The adaptability of the algorithm suggests that it could be fine-tuned for specific industry challenges, paving the way for more specialized versions of the MLBRSA. Additionally, integrating the MLBRSA with other advanced computational techniques could further enhance its capabilities. There is also scope for exploring the algorithm’s performance in dynamic environments, where problem parameters change over time. Lastly, as the world of software development continues to evolve, understanding how the MLBRSA can be integrated into modern agile and DevOps practices will be crucial.

## Figures and Tables

**Table 1:** Unimodal functions with 30-dimension results.

Functions		MLBRSA	RSA	IRSA	RLBGWO	IDMOA	LSHADE-cnEpSin	AGSK	RLAOA
**F1**	Min	**0.00E+00**	**0.00E+00**	**0.00E+00**	**0.00E+00**	1.58E-247	4.07E-124	**0.00E+00**	5.42E-256
	Max	0.00E+00	0.00E+00	1.56E-276	2.37E-165	8.34E-231	1.26E-115	0.00E+00	1.97E-239
	Avg.	0.00E+00	0.00E+00	1.98E-277	1.34E-166	5.42E-232	6.57E-117	0.00E+00	9.92E-241
	STD	0.00E+00	0.00E+00	0.00E+00	0.00E+00	0.00E+00	2.81E-116	0.00E+00	0.00E+00
**F2**	Min	**0.00E+00**	**0.00E+00**	4.88E-174	**0.00E+00**	9.35E-132	1.85E-66	3.95E-246	3.35E-127
	Max	0.00E+00	0.00E+00	5.94E-143	8.04E-74	2.05E-121	1.53E-59	9.82E-229	2.26E-119
	Avg.	0.00E+00	0.00E+00	3.57E-144	4.02E-75	1.38E-122	1.51E-60	5.65E-230	1.17E-120
	STD	0.00E+00	0.00E+00	1.32E-143	1.80E-74	4.73E-122	4.17E-60	0.00E+00	5.04E-120
**F3**	Min	**0.00E+00**	**0.00E+00**	7.05E-216	**0.00E+00**	1.05E-222	2.75E-108	**0.00E+00**	8.27E-182
	Max	0.00E+00	0.00E+00	2.59E-160	7.71E-65	1.87E-202	6.28E-98	0.00E+00	1.97E-160
	Avg.	0.00E+00	0.00E+00	1.29E-161	3.85E-66	9.34E-204	4.88E-99	0.00E+00	9.99E-162
	STD	0.00E+00	0.00E+00	5.79E-161	1.72E-65	0.00E+00	1.44E-98	0.00E+00	4.41E-161
**F4**	Min	**0.00E+00**	**0.00E+00**	6.42E-126	**0.00E+00**	7.07E-122	1.15E-59	4.60E-194	2.16E-121
	Max	0.00E+00	0.00E+00	4.39E-105	2.19E-67	5.78E-111	6.11E-53	2.08E-178	2.43E-115
	Avg.	0.00E+00	0.00E+00	3.13E-106	1.10E-68	3.04E-112	3.46E-54	1.04E-179	1.83E-116
	STD	0.00E+00	0.00E+00	1.00E-105	4.91E-68	1.29E-111	1.36E-53	0.00E+00	5.78E-116
**F5**	Min	**0.00E+00**	2.35E-25	0.00E+00	8.54E-04	2.21E+01	2.15E+01	8.21E-12	2.00E+01
	Max	0.00E+00	2.90E+01	2.87E+01	2.60E+01	2.68E+01	2.75E+01	2.57E-05	2.40E+01
	Avg.	0.00E+00	1.16E+01	1.51E+00	1.89E+01	2.40E+01	2.35E+01	2.89E-06	2.17E+01
	STD	0.00E+00	1.46E+01	6.41E+00	1.12E+01	1.25E+00	1.28E+00	6.24E-06	1.06E+00
**F6**	Min	**0.00E+00**	5.93E+00	**0.00E+00**	1.93E-07	2.36E-09	5.89E-07	8.33E-02	5.57E-09
	Max	0.00E+00	7.50E+00	0.00E+00	7.11E-05	8.77E-09	1.69E-05	2.54E-01	2.47E-01
	Avg.	0.00E+00	7.21E+00	0.00E+00	9.75E-06	4.72E-09	6.67E-06	1.60E-01	1.24E-02
	STD	0.00E+00	4.40E-01	0.00E+00	1.68E-05	1.63E-09	5.31E-06	4.13E-02	5.53E-02
**F7**	Min	5.40E-05	1.67E-06	1.54E-04	1.98E-05	5.89E-05	8.32E-05	**6.61E-07**	2.75E-05
	Max	9.14E-04	6.55E-04	2.94E-03	4.00E-03	7.83E-04	1.72E-03	1.80E-05	9.09E-04
	Avg.	3.12E-04	1.24E-04	9.44E-04	1.19E-03	2.38E-04	8.63E-04	7.95E-06	3.67E-04
	STD	2.11E-04	1.49E-04	8.21E-04	1.41E-03	1.91E-04	4.59E-04	5.66E-06	2.38E-04

**Table 2:** Multi-modal functions with 30-dimension results.

Functions		MLBRSA	RSA	IRSA	RLBGWO	IDMOA	LSHADE-cnEpSin	AGSK	RLAOA
**F8**	Min	−**1.01E+07**	−5.66E+03	−1.26E+04	−1.26E+04	−9.66E+03	−1.13E+04	−1.25E+04	−1.22E+04
	Max	−5.87E+05	−3.38E+03	−9.02E+03	−9.02E+03	−7.63E+03	−7.32E+03	−7.02E+03	−7.76E+03
	Avg.	−3.20E+06	−5.30E+03	−1.20E+04	−1.22E+04	−8.56E+03	-8.90E+03	−1.11E+04	−1.05E+04
	STD	2.48E+06	5.17E+02	1.30E+03	1.09E+03	4.99E+02	9.84E+02	1.54E+03	1.21E+03
**F9**	Min	**0.00E+00**	**0.00E+00**	**0.00E+00**	**0.00E+00**	**0.00E+00**	**0.00E+00**	**0.00E+00**	**0.00E+00**
	Max	0.00E+00	0.00E+00	0.00E+00	0.00E+00	0.00E+00	0.00E+00	0.00E+00	0.00E+00
	Avg.	0.00E+00	0.00E+00	0.00E+00	0.00E+00	0.00E+00	0.00E+00	0.00E+00	0.00E+00
	STD	0.00E+00	0.00E+00	0.00E+00	0.00E+00	0.00E+00	0.00E+00	0.00E+00	0.00E+00
**F10**	Min	**8.88E-16**	**8.88E-16**	**8.88E-16**	**8.88E-16**	**8.88E-16**	**8.88E-16**	**8.88E-16**	**8.88E-16**
	Max	8.88E-16	8.88E-16	8.88E-16	8.88E-16	8.88E-16	8.88E-16	8.88E-16	8.88E-16
	Avg.	8.88E-16	8.88E-16	8.88E-16	8.88E-16	8.88E-16	8.88E-16	8.88E-16	8.88E-16
	STD	0.00E+00	0.00E+00	0.00E+00	0.00E+00	0.00E+00	0.00E+00	0.00E+00	0.00E+00
**F11**	Min	**0.00E+00**	**0.00E+00**	**0.00E+00**	**0.00E+00**	**0.00E+00**	**0.00E+00**	**0.00E+00**	**0.00E+00**
	Max	0.00E+00	0.00E+00	0.00E+00	0.00E+00	0.00E+00	0.00E+00	0.00E+00	0.00E+00
	Avg.	0.00E+00	0.00E+00	0.00E+00	0.00E+00	0.00E+00	0.00E+00	0.00E+00	0.00E+00
	STD	0.00E+00	0.00E+00	0.00E+00	0.00E+00	0.00E+00	0.00E+00	0.00E+00	0.00E+00
**F12**	Min	**1.57E-32**	6.00E-01	**1.57E-32**	1.83E-08	1.75E-09	7.08E-08	1.38E-03	6.34E-09
	Max	1.57E-32	1.67E+00	1.57E-32	3.67E-07	6.23E-09	1.04E-01	3.76E-03	6.53E-03
	Avg.	1.57E-32	1.41E+00	1.57E-32	1.29E-07	3.64E-09	5.18E-03	2.38E-03	1.29E-03
	STD	2.81E-48	3.69E-01	2.81E-48	8.58E-08	1.16E-09	2.32E-02	6.54E-04	2.66E-03
**F13**	Min	**1.35E-32**	1.90E-30	**1.35E-32**	8.74E-09	3.10E-08	2.22E-06	2.37E-11	5.24E-10
	Max	1.35E-32	3.00E+00	2.97E+00	6.96E-06	5.48E-02	6.48E-02	2.45E-05	3.27E-01
	Avg.	1.35E-32	6.00E-01	3.90E-01	1.74E-06	1.41E-02	1.26E-02	2.04E-06	3.06E-02
	STD	2.81E-48	1.23E+00	7.56E-01	1.94E-06	1.58E-02	1.91E-02	6.17E-06	7.23E-02

**Table 3:** Multi-modal functions with fixed dimensions.

Functions		MLBRSA	RSA	IRSA	RLBGWO	IDMOA	LSHADE-cnEpSin	AGSK	RLAOA
**F14**	Min	**9.98E-01**	**9.98E-01**	**9.98E-01**	**9.98E-01**	**9.98E-01**	**9.98E-01**	**9.98E-01**	**9.98E-01**
	Max	9.98E-01	1.08E+01	1.99E+00	9.98E-01	2.98E+00	1.99E+00	2.98E+00	1.08E+01
	Avg.	9.98E-01	4.32E+00	1.05E+00	9.98E-01	1.15E+00	1.10E+00	1.25E+00	2.53E+00
	STD	0.00E+00	3.38E+00	2.22E-01	1.69E-16	4.86E-01	3.06E-01	5.46E-01	2.35E+00
**F15**	Min	**3.07E-04**	6.74E-04	**3.07E-04**	3.08E-04	**3.07E-04**	**3.07E-04**	**3.07E-04**	**3.07E-04**
	Max	3.07E-04	5.72E-03	3.07E-04	1.60E-03	1.22E-03	2.04E-02	4.27E-04	3.07E-04
	Avg.	3.07E-04	2.83E-03	3.07E-04	6.25E-04	6.74E-04	1.51E-03	3.14E-04	3.07E-04
	STD	3.20E-19	1.50E-03	3.57E-19	3.46E-04	4.60E-04	4.46E-03	2.67E-05	6.90E-19
**F16**	Min	**−1.0316**	**−1.0316**	**−1.0316**	**−1.0316**	**−1.0316**	**−1.0316**	**−1.0316**	**−1.0316**
	Max	−1.0316	−1.0282	−1.0316	−1.0316	−1.0316	−1.0316	−1.0316	−1.0316
	Avg.	−1.0316	−1.0308	−1.0316	−1.0316	−1.0316	−1.0316	−1.0316	−1.0316
	STD	1.99E-08	9.54E-04	2.28E-16	2.10E-16	1.84E-16	1.97E-16	4.74E-08	2.10E-16
**F17**	Min	**0.39789**	**0.39789**	**0.39789**	**0.39789**	**0.39789**	**0.39789**	**0.39789**	**0.39789**
	Max	0.39789	0.39790	0.39789	0.39789	0.39789	0.39789	0.39789	0.39789
	Avg.	0.39789	0.39789	0.39789	0.39789	0.39789	0.39789	0.39789	0.39789
	STD	1.79E-14	2.64E-06	0.00E+00	0.00E+00	0.00E+00	0.00E+00	1.68E-06	0.00E+00
**F18**	Min	**3.0000**	**3.0000**	**3.0000**	**3.0000**	**3.0000**	**3.0000**	**3.0000**	**3.0000**
	Max	3.0000	3.0023	3.0000	3.0000	3.0000	3.0000	3.0000	84.0000
	Avg.	3.0000	3.0003	3.0000	3.0000	3.0000	3.0000	3.0000	7.0500
	STD	8.46E-16	6.09E-04	1.03E-15	3.05E-15	3.10E-15	1.30E-15	4.46E-06	1.81E+01
**F19**	Min	**−3.8628**	**−3.8261**	−3.8628	**−3.8628**	**−3.8628**	**−3.8628**	**−3.8628**	**−3.8628**
	Max	−3.8628	−3.6173	−3.8628	−3.8628	−3.8628	−3.8628	−3.8628	−3.8628
	Avg.	−3.8628	−3.7714	−3.8628	−3.8628	−3.8628	−3.8628	−3.8628	−3.8628
	STD	2.24E-15	5.26E-02	2.28E-15	2.26E-15	2.00E-15	2.28E-15	3.67E-06	2.24E-15
**F20**	Min	−**3.3220**	−3.1997	**−3.3220**	**−3.3220**	**−3.3220**	**−3.3220**	**−3.3220**	**−3.3220**
	Max	−3.2031	−1.4209	−3.2031	−3.1327	−3.2031	−3.2031	−3.2007	−3.2031
	Avg.	−3.2507	−2.6042	−3.2982	−3.2736	−3.2447	−3.2566	−3.2980	−3.2744
	STD	5.98E-02	4.06E-01	4.88E-02	7.02E-02	5.82E-02	6.07E-02	4.91E-02	5.98E-02
**F21**	Min	**−10.1532**	−5.0552	−5.0552	**−10.1532**	**−10.1532**	**−10.1532**	−10.1531	**−10.1532**
	Max	−10.1532	−5.0552	−5.0552	−5.0552	−5.0552	−2.6305	−10.1513	−2.6305
	Avg.	−10.1532	−5.0552	−5.0552	−9.3885	−9.8983	−7.4830	−10.1526	−7.4830
	STD	3.05E-15	2.80E-07	0.00E+00	1.87E+00	1.14E+00	2.79E+00	4.56E-04	2.79E+00
**F22**	Min	**−10.4029**	−5.0877	−5.0877	**−10.4029**	**−10.4029**	**−10.4029**	**−10.4029**	**−10.4029**
	Max	−10.4029	−5.0877	−5.0877	−5.0877	−5.0877	−5.0877	−10.4009	−1.8376
	Avg.	−10.4029	−5.0877	−5.0877	−9.8714	−9.8714	−8.0111	−10.4021	−7.7122
	STD	3.36E-15	9.43E-07	1.93E-15	1.64E+00	1.64E+00	2.71E+00	6.10E-04	3.14E+00
**F23**	Min	**−10.5364**	−5.1285	**−10.5364**	**−10.5364**	**−10.5364**	**−10.5364**	−10.5362	**−10.5364**
	Max	−10.5364	−5.1285	−5.1285	−10.5363	−5.1285	−5.1285	−10.5342	−1.6766
	Avg.	−10.5364	−5.1285	−5.6693	−10.5364	−9.7252	−8.3732	−10.5356	−7.8007
	STD	2.97E-15	2.01E-06	1.66E+00	2.03E-05	1.98E+00	2.72E+00	5.13E-04	3.54E+00

**Table 4:** Results obtained for the welded beam problem.

Algorithm	h	l	t	b	Min	Max	Avg.	STD	RT	FRT
MLBRSA	0.2057	3.2530	9.0366	0.2057	1.695E+00	1.695E+00	1.695E+00	3.014E-16	0.49	1.38
IRSA	0.2057	3.2530	9.0366	0.2057	1.695E+00	1.695E+00	1.695E+00	4.874E-09	0.73	4.05
RSA	0.2057	3.2530	9.0366	0.2057	1.695E+00	1.695E+00	1.695E+00	3.374E-06	0.11	4.65
RLBGWO	0.2057	3.2530	9.0366	0.2057	1.695E+00	1.703E+00	1.696E+00	1.860E-03	1.08	6.80
IDMOA	0.2057	3.2531	9.0366	0.2057	1.695E+00	1.790E+00	1.716E+00	3.029E-02	0.68	7.80
LSHADE-cnEpSin	0.2057	3.2530	9.0366	0.2057	1.695E+00	1.695E+00	1.695E+00	3.602E-16	0.84	1.63
AGSK	0.2057	3.2530	9.0366	0.2057	1.695E+00	1.695E+00	1.695E+00	2.211E-05	0.53	4.90
RLAOA	0.2057	3.2530	9.0366	0.2057	1.695E+00	1.695E+00	1.695E+00	4.925E-05	0.62	4.80

**Table 5:** Results obtained for the pressure vessel design problem.

Algorithm	$T_{s}$	$T_{h}$	R	L	Min	Max	Avg.	STD	RT	FRT
MLBRSA	1.094	2.94E-18	65.225	10.000	2.303E+03	2.303E+03	2.303E+03	0.000E+00	0.18	1.00
IRSA	1.094	0.00E+00	65.225	10.000	2.303E+03	2.303E+03	2.303E+03	3.460E-13	0.81	4.50
RSA	1.094	5.01E-23	65.225	10.000	2.303E+03	2.303E+03	2.303E+03	0.000E+00	0.08	3.55
RLBGWO	1.094	0.00E+00	65.225	10.000	2.303E+03	6.055E+03	2.876E+03	1.188E+03	0.57	6.35
IDMOA	1.094	6.12E-18	65.225	10.000	2.303E+03	2.303E+03	2.303E+03	3.904E-13	0.34	4.83
LSHADE-cnEpSin	2.768	−4.51E+01	205.691	9.997	−3.347E+06	−2.478E+05	−1.368E+06	7.297E+05	0.85	6.83
AGSK	1.094	1.30E-17	65.225	10.000	2.303E+03	3.624E+03	2.567E+03	5.424E+02	0.28	3.55
RLAOA	1.094	3.57E-19	65.225	10.000	2.303E+03	3.637E+03	2.766E+03	6.477E+02	0.37	5.40

**Table 6:** Results obtained for the tension/compression spring design problem.

Algorithm	d	D	P	Min	Max	Avg.	STD	RT	FRT
MLBRSA	0.1391	1.3000	11.8924	3.662E+00	3.662E+00	3.662E+00	8.087E-16	0.23	2.08
IRSA	0.1391	1.3000	11.8924	3.662E+00	3.662E+00	3.662E+00	1.248E-15	0.85	6.70
RSA	0.1391	1.3000	11.8924	3.662E+00	3.662E+00	3.662E+00	1.197E-15	0.10	3.90
RLBGWO	0.1391	1.3000	11.8924	3.662E+00	3.662E+00	3.662E+00	1.317E-15	0.61	6.85
IDMOA	0.1391	1.3000	11.8924	3.662E+00	3.662E+00	3.662E+00	2.213E-15	0.54	6.10
LSHADE-cnEpSin	0.1413	1.3626	10.9903	3.639E+00	3.639E+00	3.639E+00	6.040E-09	0.80	1.00
AGSK	0.1391	1.3000	11.8924	3.662E+00	3.662E+00	3.662E+00	1.305E-15	0.31	4.43
RLAOA	0.1391	1.3000	11.8924	3.662E+00	3.662E+00	3.662E+00	1.462E-15	0.37	4.95

**Table 7:** Results obtained for the three-bar truss design problem.

Algorithm	x1	x2	Min	Max	Avg.	STD	RT	FRT
MLBRSA	0.78685	0.28801	1.864E+02	1.864E+02	1.864E+02	5.832E-14	0.14	2.55
IRSA	0.78685	0.28801	1.864E+02	1.864E+02	1.864E+02	4.829E-12	0.85	8.00
RSA	0.78685	0.28801	1.864E+02	1.864E+02	1.864E+02	7.434E-14	0.08	4.25
RLBGWO	0.78685	0.28801	1.864E+02	1.864E+02	1.864E+02	6.520E-14	0.56	5.15
IDMOA	0.78685	0.28801	1.864E+02	1.864E+02	1.864E+02	8.118E-14	0.46	4.38
LSHADE-cnEpSin	0.78685	0.28801	1.864E+02	1.864E+02	1.864E+02	5.609E-14	0.50	2.90
AGSK	0.78685	0.28801	1.864E+02	1.864E+02	1.864E+02	7.290E-14	0.28	4.40
RLAOA	0.78685	0.28801	1.864E+02	1.864E+02	1.864E+02	7.290E-14	0.37	4.38

**Table 8:** Results obtained for the tubular column design problem.

Algorithm	d	t	Min	Max	Avg.	STD	RT	FRT
MLBRSA	5.45273	0.29154	2.649E+01	2.649E+01	2.649E+01	7.290E-15	0.38	4.03
IRSA	5.45273	0.29154	2.649E+01	2.649E+01	2.649E+01	7.290E-15	0.82	4.03
RSA	5.45273	0.29154	2.649E+01	2.649E+01	2.649E+01	7.514E-15	0.55	4.23
RLBGWO	5.45273	0.29154	2.649E+01	2.649E+01	2.649E+01	8.816E-15	0.07	6.43
IDMOA	5.45273	0.29154	2.649E+01	2.649E+01	2.649E+01	1.031E-14	0.15	5.23
LSHADE-cnEpSin	5.45273	0.29154	2.649E+01	2.649E+01	2.649E+01	7.290E-15	0.56	4.03
AGSK	5.45273	0.29154	2.649E+01	2.649E+01	2.649E+01	7.290E-15	0.28	4.03
RLAOA	5.45273	0.29154	2.649E+01	2.649E+01	2.649E+01	7.290E-15	0.37	4.03

## Data Availability

All other data is included in the paper, and no additional data has been used in this study.

## Appendix A

**Table A1:** Various parameters of the selected algorithms.

S. No.	Algorithm	Parameters
1	RSA	α=0.1 and β=0.005
2	MLBRSA	α and β are adaptable, Exploration rate ξ=0.1, Learning rate for QL λ=0.5, Discount factor γ=0.9
3	RLBGWO	Exploration rate ξ=0.1, Learning rate for QL λ=0.5, Discount factor γ=0.9
4	IDMOA	phi=[−1, 1], CF=[0, 1]
5	IRSA	α=0.1, β=0.1, kn=9, and ke=T /10
6	LSHADE-cnEpSin	μF=0.5, μCR=0.5, μfreq=0.5, H=5, freq=0.5, ps=0.5, and pc=0.4
7	AGSK	p=0.05, 5% of N is suitable for the best partition size is 5% , $worst\;partition\;size\;is\;5\%\;,and\;the\;middle\;partition\;size\;is\;90\%,KwP=\left[0.85,0.05,0.05,0.05\right],\;and\;c=0.05.$
8	RLAOA	m=10, n=2, ξ linearly increasing from 0 to 0.9, α=0.1, ω=0.01, $\gamma =0.9,\;\lambda =5,\;\mu =0.499,\;s=0.01,\;r^{\sim }=10,\;\alpha =0.1,\;\beta =1.5,$ and Max episodes=100

**Table A2:** Test functions F1–F13 with 100 dimensions.

Functions		MLBRSA	RSA	IRSA	RLBGWO	IDMOA	LSHADE-cnEpSin	AGSK	RLAOA
**F1**	Min	**0.00E+00**	**0.00E+00**	**0.00E+00**	**0.00E+00**	1.18E-241	6.58E-123	**0.00E+00**	2.66E-247
	Max	0.00E+00	0.00E+00	1.65E-257	1.97E-144	3.27E-221	1.15E-109	0.00E+00	3.95E-233
	Avg.	0.00E+00	0.00E+00	8.23E-259	9.85E-146	1.63E-222	5.75E-111	0.00E+00	1.98E-234
	STD	0.00E+00	0.00E+00	0.00E+00	4.40E-145	0.00E+00	2.56E-110	0.00E+00	0.00E+00
**F2**	Min	**0.00E+00**	**0.00E+00**	7.08E-161	**0.00E+00**	3.65E-128	1.78E-62	7.13E-234	6.78E-125
	Max	0.00E+00	0.00E+00	4.37E-133	1.37E-70	2.51E-117	9.31E-57	7.80E-217	9.99E-116
	Avg.	0.00E+00	0.00E+00	2.19E-134	6.85E-72	1.45E-118	4.95E-58	4.01E-218	5.21E-117
	STD	0.00E+00	0.00E+00	9.77E-134	3.06E-71	5.59E-118	2.08E-57	0.00E+00	2.23E-116
**F3**	Min	**0.00E+00**	**0.00E+00**	3.76E-178	**0.00E+00**	7.40E-215	4.03E-101	**0.00E+00**	2.53E-154
	Max	0.00E+00	0.00E+00	6.21E-147	3.51E-28	7.59E-193	1.60E-87	0.00E+00	1.01E-135
	Avg.	0.00E+00	0.00E+00	3.14E-148	1.92E-29	3.79E-194	8.46E-89	0.00E+00	5.10E-137
	STD	0.00E+00	0.00E+00	1.39E-147	7.86E-29	0.00E+00	3.58E-88	0.00E+00	2.26E-136
**F4**	Min	**0.00E+00**	**0.00E+00**	7.64E-117	**0.00E+00**	1.77E-119	4.20E-55	8.14E-175	2.75E-119
	Max	0.00E+00	0.00E+00	3.93E-87	3.11E-64	1.66E-104	3.55E-47	2.33E-155	4.36E-112
	Avg.	0.00E+00	0.00E+00	1.97E-88	1.56E-65	8.31E-106	1.87E-48	1.16E-156	3.78E-113
	STD	0.00E+00	0.00E+00	8.80E-88	6.96E-65	3.71E-105	7.92E-48	5.21E-156	1.03E-112
**F5**	Min	**0.00E+00**	9.90E+01	9.24E+01	4.61E-03	9.30E+01	9.39E+01	1.07E-10	9.12E+01
	Max	0.00E+00	9.90E+01	9.81E+01	9.80E+01	9.82E+01	9.82E+01	2.72E-05	9.63E+01
	Avg.	0.00E+00	9.90E+01	9.42E+01	6.24E+01	9.53E+01	9.67E+01	2.41E-06	9.36E+01
	STD	0.00E+00	0.00E+00	1.63E+00	4.69E+01	1.74E+00	1.46E+00	6.11E-06	1.27E+00
**F6**	Min	**0.00E+00**	2.50E+01	0.00E+00	1.07E-05	2.18E-04	7.51E-02	2.26E+00	2.53E-03
	Max	0.00E+00	2.50E+01	0.00E+00	1.99E-03	1.31E-02	4.77E-01	4.02E+00	3.80E+00
	Avg.	0.00E+00	2.50E+01	0.00E+00	4.68E-04	2.99E-03	2.11E-01	3.26E+00	1.85E+00
	STD	0.00E+00	0.00E+00	0.00E+00	6.62E-04	3.88E-03	8.50E-02	4.66E-01	9.94E-01
**F7**	Min	5.01E-05	3.05E-06	1.06E-04	7.16E-05	8.22E-05	3.31E-05	**2.31E-06**	4.73E-05
	Max	9.97E-04	3.72E-04	2.44E-03	1.06E-02	1.08E-03	2.41E-03	3.61E-05	1.80E-03
	Avg.	3.25E-04	9.96E-05	8.96E-04	1.88E-03	4.09E-04	9.12E-04	1.75E-05	4.31E-04
	STD	2.35E-04	8.64E-05	6.33E-04	2.95E-03	2.73E-04	6.43E-04	1.12E-05	3.92E-04
**F8**	Min	**2.36E+08**	−1.77E+04	−4.19E+04	−4.19E+04	−3.27E+04	−3.21E+04	−3.78E+04	−4.12E+04
	Max	−1.47E+06	−1.43E+04	−2.15E+04	−3.01E+04	−2.37E+04	−2.46E+04	−2.38E+04	−2.51E+04
	Avg.	−1.81E+07	−1.60E+04	−3.55E+04	−4.13E+04	−2.81E+04	−2.80E+04	−2.89E+04	−3.68E+04
	STD	5.16E+07	9.70E+02	6.77E+03	2.64E+03	2.36E+03	1.67E+03	4.03E+03	4.74E+03
**F9**	Min	**0.00E+00**	**0.00E+00**	**0.00E+00**	**0.00E+00**	**0.00E+00**	**0.00E+00**	**0.00E+00**	**0.00E+00**
	Max	0.00E+00	0.00E+00	0.00E+00	0.00E+00	0.00E+00	0.00E+00	0.00E+00	0.00E+00
	Avg.	0.00E+00	0.00E+00	0.00E+00	0.00E+00	0.00E+00	0.00E+00	0.00E+00	0.00E+00
	STD	0.00E+00	0.00E+00	0.00E+00	0.00E+00	0.00E+00	0.00E+00	0.00E+00	0.00E+00
**F10**	Min	**8.88E-16**	**8.88E-16**	**8.88E-16**	**8.88E-16**	**8.88E-16**	**8.88E-16**	**8.88E-16**	**8.88E-16**
	Max	8.88E-16	8.88E-16	8.88E-16	8.88E-16	8.88E-16	8.88E-16	8.88E-16	8.88E-16
	Avg.	8.88E-16	8.88E-16	8.88E-16	8.88E-16	8.88E-16	8.88E-16	8.88E-16	8.88E-16
	STD	0.00E+00	0.00E+00	0.00E+00	0.00E+00	0.00E+00	0.00E+00	0.00E+00	0.00E+00
**F11**	Min	**0.00E+00**	**0.00E+00**	**0.00E+00**	**0.00E+00**	**0.00E+00**	**0.00E+00**	**0.00E+00**	**0.00E+00**
	Max	0.00E+00	0.00E+00	0.00E+00	0.00E+00	0.00E+00	0.00E+00	0.00E+00	0.00E+00
	Avg.	0.00E+00	0.00E+00	0.00E+00	0.00E+00	0.00E+00	0.00E+00	0.00E+00	0.00E+00
	STD	0.00E+00	0.00E+00	0.00E+00	0.00E+00	0.00E+00	0.00E+00	0.00E+00	0.00E+00
**F12**	Min	**4.71E-33**	1.27E+00	**4.71E-33**	1.52E-09	5.40E-06	1.53E-03	1.15E-05	4.92E-03
	Max	4.71E-33	1.33E+00	4.71E-33	5.99E-05	9.76E-05	6.18E-03	5.16E-02	6.32E-02
	Avg.	4.71E-33	1.32E+00	4.71E-33	8.49E-06	3.60E-05	2.66E-03	2.28E-02	2.33E-02
	STD	1.40E-48	1.14E-02	1.40E-48	1.55E-05	2.09E-05	1.13E-03	1.98E-02	1.44E-02
**F13**	Min	**1.35E-32**	5.65E+00	**1.35E-32**	2.42E-07	8.09E-03	4.48E-01	1.07E-12	1.35E-01
	Max	1.35E-32	1.00E+01	9.89E+00	7.19E+00	3.57E-01	8.34E+00	1.28E-07	1.50E+00
	Avg.	1.35E-32	9.78E+00	6.79E+00	3.60E-01	6.90E-02	4.36E+00	1.16E-08	6.63E-01
	STD	2.81E-48	9.73E-01	3.08E+00	1.61E+00	7.78E-02	3.55E+00	2.90E-08	3.79E-01

**Table A3:** Test functions F1–F13 with 500 dimensions.

Functions		MLBRSA	RSA	IRSA	RLBGWO	IDMOA	LSHADE-cnEpSin	AGSK	RLAOA
**F1**	Min	**0.00E+00**	**0.00E+00**	2.09E-281	0.00E+00	1.61E-231	5.32E-117	**0.00E+00**	5.22E-237
	Max	0.00E+00	0.00E+00	3.58E-246	2.78E-140	9.02E-218	2.71E-105	0.00E+00	1.63E-226
	Avg.	0.00E+00	0.00E+00	2.84E-247	1.85E-141	6.01E-219	2.05E-106	0.00E+00	1.09E-227
	STD	0.00E+00	0.00E+00	0.00E+00	7.18E-141	0.00E+00	6.94E-106	0.00E+00	0.00E+00
**F2**	Min	**0.00E+00**	**0.00E+00**	6.50E-149	2.02E+01	3.97E-124	8.59E-61	7.70E-233	3.59E-121
	Max	2.51E-270	0.00E+00	4.67E-123	2.82E+269	9.90E-115	1.30E-55	1.92E-209	8.34E-116
	Avg.	1.67E-271	0.00E+00	3.12E-124	1.88E+268	6.97E-116	1.53E-56	2.50E-210	7.58E-117
	STD	0.00E+00	0.00E+00	1.21E-123	6.55E+04	2.55E-115	3.47E-56	0.00E+00	2.18E-116
**F3**	Min	**0.00E+00**	**0.00E+00**	3.21E-217	**0.00E+00**	2.67E-208	7.50E-94	**0.00E+00**	1.08E-115
	Max	0.00E+00	0.00E+00	1.30E-119	9.84E+02	2.16E-189	4.66E-81	3.60E-308	2.86E-93
	Avg.	0.00E+00	0.00E+00	8.66E-121	7.02E+01	1.54E-190	3.35E-82	0.00E+00	1.99E-94
	STD	0.00E+00	0.00E+00	3.35E-120	2.54E+02	0.00E+00	1.20E-81	0.00E+00	7.36E-94
**F4**	Min	**0.00E+00**	**0.00E+00**	2.45E-95	**0.00E+00**	2.40E-114	1.22E-49	4.30E-154	1.00E-114
	Max	0.00E+00	0.00E+00	2.29E-78	1.87E-70	1.03E-103	8.21E-43	3.04E-141	4.48E-104
	Avg.	0.00E+00	0.00E+00	1.90E-79	1.29E-71	8.97E-105	1.46E-43	2.05E-142	4.95E-105
	STD	0.00E+00	0.00E+00	5.99E-79	4.82E-71	2.71E-104	2.85E-43	7.85E-142	1.29E-104
**F5**	Min	**0.00E+00**	4.99E+02	4.91E+02	2.72E-01	4.92E+02	4.95E+02	6.76E-08	4.92E+02
	Max	0.00E+00	4.99E+02	4.96E+02	4.94E+02	4.95E+02	4.96E+02	6.79E-05	4.95E+02
	Avg.	0.00E+00	4.99E+02	4.92E+02	3.62E+02	4.94E+02	4.96E+02	7.87E-06	4.94E+02
	STD	0.00E+00	0.00E+00	1.28E+00	2.26E+02	1.11E+00	5.21E-01	1.84E-05	8.24E-01
**F6**	Min	**0.00E+00**	1.25E+02	0.00E+00	3.46E-07	1.59E+00	1.84E+01	8.98E+01	2.04E+01
	Max	0.00E+00	1.25E+02	0.00E+00	2.14E-02	2.11E+00	3.05E+01	9.89E+01	3.62E+01
	Avg.	0.00E+00	1.25E+02	0.00E+00	3.69E-03	1.90E+00	2.37E+01	9.36E+01	2.94E+01
	STD	0.00E+00	0.00E+00	0.00E+00	6.34E-03	1.50E-01	4.08E+00	2.33E+00	3.85E+00
**F7**	Min	1.17E-05	5.08E-05	5.13E-05	1.35E-05	3.16E-05	1.81E-04	**8.67E-06**	4.41E-05
	Max	6.98E-04	4.50E-04	1.60E-03	8.80E-03	9.33E-04	3.78E-03	7.36E-05	1.29E-03
	Avg.	3.31E-04	2.10E-04	5.80E-04	1.14E-03	3.03E-04	1.36E-03	3.42E-05	4.35E-04
	STD	2.14E-04	1.23E-04	4.48E-04	2.22E-03	2.82E-04	9.87E-04	2.07E-05	3.11E-04
**F8**	Min	−**1.24E+08**	−7.87E+04	−2.09E+05	−2.09E+05	−1.32E+05	−1.30E+05	−2.04E+05	−2.07E+05
	Max	−4.48E+06	−5.26E+04	−1.50E+05	−1.48E+05	−9.55E+04	−7.89E+04	−4.25E+04	−1.55E+05
	Avg.	−3.17E+07	−6.25E+04	−1.90E+05	−2.05E+05	−1.21E+05	−1.07E+05	−1.30E+05	−1.81E+05
	STD	3.38E+07	6.75E+03	2.89E+04	1.56E+04	8.81E+03	1.56E+04	4.25E+04	1.89E+04
**F9**	Min	**0.00E+00**	**0.00E+00**	**0.00E+00**	**0.00E+00**	**0.00E+00**	**0.00E+00**	**0.00E+00**	**0.00E+00**
	Max	0.00E+00	0.00E+00	0.00E+00	0.00E+00	0.00E+00	0.00E+00	0.00E+00	0.00E+00
	Avg.	0.00E+00	0.00E+00	0.00E+00	0.00E+00	0.00E+00	0.00E+00	0.00E+00	0.00E+00
	STD	0.00E+00	0.00E+00	0.00E+00	0.00E+00	0.00E+00	0.00E+00	0.00E+00	0.00E+00
**F10**	Min	**8.88E-16**	**8.88E-16**	**8.88E-16**	**8.88E-16**	**8.88E-16**	**8.88E-16**	**8.88E-16**	**8.88E-16**
	Max	8.88E-16	8.88E-16	8.88E-16	8.88E-16	8.88E-16	8.88E-16	8.88E-16	8.88E-16
	Avg.	8.88E-16	8.88E-16	8.88E-16	8.88E-16	8.88E-16	8.88E-16	8.88E-16	8.88E-16
	STD	0.00E+00	0.00E+00	0.00E+00	0.00E+00	0.00E+00	0.00E+00	0.00E+00	0.00E+00
**F11**	Min	**0.00E+00**	**0.00E+00**	**0.00E+00**	**0.00E+00**	**0.00E+00**	**0.00E+00**	**0.00E+00**	**0.00E+00**
	Max	0.00E+00	0.00E+00	0.00E+00	0.00E+00	0.00E+00	0.00E+00	0.00E+00	0.00E+00
	Avg.	0.00E+00	0.00E+00	0.00E+00	0.00E+00	0.00E+00	0.00E+00	0.00E+00	0.00E+00
	STD	0.00E+00	0.00E+00	0.00E+00	0.00E+00	0.00E+00	0.00E+00	0.00E+00	0.00E+00
**F12**	Min	**4.71E-33**	1.27E+00	**4.71E-33**	1.52E-09	5.40E-06	1.53E-03	1.15E-05	4.92E-03
	Max	4.71E-33	1.33E+00	4.71E-33	5.99E-05	9.76E-05	6.18E-03	5.16E-02	6.32E-02
	Avg.	4.71E-33	1.32E+00	4.71E-33	8.49E-06	3.60E-05	2.66E-03	2.28E-02	2.33E-02
	STD	1.40E-48	1.14E-02	1.40E-48	1.55E-05	2.09E-05	1.13E-03	1.98E-02	1.44E-02
**F13**	Min	**1.35E-32**	5.65E+00	**1.35E-32**	2.42E-07	8.09E-03	4.48E-01	1.07E-12	1.35E-01
	Max	1.35E-32	1.00E+01	9.89E+00	7.19E+00	3.57E-01	8.34E+00	1.28E-07	1.50E+00
	Avg.	1.35E-32	9.78E+00	6.79E+00	3.60E-01	6.90E-02	4.36E+00	1.16E-08	6.63E-01
	STD	2.81E-48	9.73E-01	3.08E+00	1.61E+00	7.78E-02	3.55E+00	2.90E-08	3.79E-01

**Table A4:** RT values for 23 test functions.

Problem	MLBRSA	IRSA	RSA	RLBGWO	IDMOA	LSHADE-cnEpSin	AGSK	RLAOA
F1	0.28	0.31	0.11	0.50	4.43	0.74	1.78	0.77
F2	0.20	0.27	0.07	0.39	4.28	0.64	1.38	0.49
F3	0.22	0.24	0.11	0.47	8.84	0.52	1.77	0.46
F4	0.13	0.17	0.04	0.24	2.64	0.44	0.72	0.30
F5	0.13	0.16	0.05	0.27	3.12	0.41	0.97	0.38
F6	0.17	0.22	0.05	0.32	3.51	0.60	1.11	0.43
F7	0.23	0.28	0.10	0.51	8.32	0.63	1.75	0.55
F8	0.20	0.24	0.07	0.40	4.95	0.63	1.16	0.49
F9	0.19	0.23	0.12	0.37	4.11	0.60	1.12	0.47
F10	0.19	0.23	0.11	0.36	4.38	0.63	0.92	0.48
F11	0.20	0.25	0.15	0.40	5.15	0.63	1.15	0.49
F12	0.33	0.39	0.19	0.82	16.00	0.75	2.76	0.74
F13	0.34	0.39	0.18	0.82	15.95	0.75	2.69	0.73
F14	0.36	0.40	0.24	1.02	22.11	0.28	2.58	0.76
F15	0.10	0.14	0.02	0.22	1.93	0.07	0.39	0.27
F16	0.09	0.12	0.02	0.20	1.67	0.05	0.35	0.27
F17	0.11	0.14	0.02	0.24	1.58	0.02	0.44	0.41
F18	0.13	0.15	0.02	0.24	1.52	0.05	0.43	0.35
F19	0.12	0.16	0.03	0.27	2.41	0.08	0.49	0.34
F20	0.11	0.12	0.02	0.21	2.11	0.10	0.41	0.32
F21	0.13	0.16	0.03	0.27	2.71	0.10	0.52	0.35
F22	0.10	0.13	0.03	0.24	2.73	0.09	0.48	0.30
F23	0.11	0.14	0.03	0.25	3.13	0.09	0.50	0.31
**Mean RT**	0.18	0.22	**0.08**	0.39	5.55	0.39	1.12	0.45
**Rank**	2	3	**1**	4.5	8	4.5	7	6

**Table A5:** FRT values for 23 benchmark functions.

Problem	MLBRSA	RSA	IRSA	RLBGWO	IDMOA	LSHADE-cnEpSin	AGSK	RLAOA
F1	2.300	2.300	4.600	4.300	6.400	8.000	2.300	5.800
F2	1.775	1.775	4.550	4.250	5.750	8.000	3.550	6.350
F3	2.175	2.175	5.350	5.875	4.400	7.550	2.175	6.300
F4	1.600	1.600	5.900	6.000	5.200	8.000	3.200	4.500
F5	1.350	4.850	2.250	6.500	6.350	6.250	3.450	5.000
F6	1.500	8.000	1.500	5.400	3.000	5.350	6.950	4.300
F7	4.800	2.950	6.050	5.300	4.150	6.750	1.050	4.950
F8	1.000	8.000	2.500	3.050	6.400	6.000	4.250	4.800
F9	4.500	4.500	4.500	4.500	4.500	4.500	4.500	4.500
F10	4.500	4.500	4.500	4.500	4.500	4.500	4.500	4.500
F11	4.500	4.500	4.500	4.500	4.500	4.500	4.500	4.500
F12	1.500	8.000	1.500	4.600	3.000	5.650	6.750	5.000
F13	1.325	3.750	3.675	4.950	5.900	6.550	3.800	6.050
F14	2.725	7.750	2.925	3.350	4.600	3.125	6.450	5.075
F15	2.225	7.850	2.300	6.300	5.500	3.725	5.450	2.650
F16	6.050	8.000	2.550	2.925	3.425	3.175	6.950	2.925
F17	3.625	7.600	3.475	3.475	3.475	3.475	7.400	3.475
F18	2.225	7.850	2.875	3.750	4.525	3.150	7.050	4.575
F19	3.350	8.000	3.075	3.225	4.900	3.075	7.000	3.375
F20	4.025	8.000	2.425	3.725	5.575	3.825	4.650	3.775
F21	1.600	7.900	6.300	3.225	3.550	4.250	4.800	4.375
F22	1.975	7.850	6.325	2.625	4.150	3.900	4.900	4.275
F23	2.150	7.750	5.900	2.900	4.200	3.875	5.150	4.075
**Mean FRT**	**2.729**	5.889	3.892	4.314	4.693	5.095	4.816	4.571
**Rank**	**1**	8	2	3	5	7	6	4
